# Lessons on Drug Development: A Literature Review of Challenges Faced in Nonalcoholic Fatty Liver Disease (NAFLD) Clinical Trials

**DOI:** 10.3390/ijms24010158

**Published:** 2022-12-21

**Authors:** Joel Yeh Siang Chen, Damien Chua, Carissa Odelia Lim, Wan Xi Ho, Nguan Soon Tan

**Affiliations:** 1 Lee Kong Chian School of Medicine, Nanyang Technological University Singapore, 11 Mandalay Road, Singapore 308232, Singapore; 2 School of Biological Sciences, Nanyang Technological University Singapore, 60 Nanyang Drive, Singapore 637551, Singapore

**Keywords:** NAFLD, NASH, NAFLD diagnostics, therapeutics, NAFLD clinical management, oral hypoglycemic agents (OHGA), diabetic drugs, clinical trials, PPAR agonists, bile acid pathway drugs

## Abstract

NAFLD is the most common chronic liver disease worldwide, occurring in both obese and lean patients. It can lead to life-threatening liver diseases and nonhepatic complications, such as cirrhosis and cardiovascular diseases, that burden public health and the health care system. Current care is weight loss through diet and exercise, which is a challenging goal to achieve. However, there are no FDA-approved pharmacotherapies for NAFLD. This review thoroughly examines the clinical trial findings from 22 drugs (Phase 2 and above) and evaluates the future direction that trials should take for further drug development. These trialed drugs can broadly be categorized into five groups—hypoglycemic, lipid-lowering, bile-pathway, anti-inflammatory, and others, which include nutraceuticals. The multitude of challenges faced in these yet-to-be-approved NAFLD drug trials provided insight into a few areas of improvement worth considering. These include drug repurposing, combinations, noninvasive outcomes, standardization, adverse event alleviation, and the need for precision medicine with more extensive consideration of NAFLD heterogenicity in drug trials. Understandably, every evolution of the drug development landscape lies with its own set of challenges. However, this paper believes in the importance of always learning from lessons of the past, with each potential improvement pushing clinical trials an additional step forward toward discovering appropriate drugs for effective NAFLD management.

## 1. Introduction

### 1.1. Prevalence of NAFLD

In the past 20 years, nonalcoholic fatty liver disease (NAFLD) has become the most common chronic liver disease and one of the biggest contributors to cirrhosis. NAFLD prevalence worldwide is at 30%, increasing in tandem with well-established risk factors such as type 2 diabetes (T2DM) and obesity [1]. The Middle East (32%) and South America (30%) have the highest prevalence, followed by Asia (27%), with a disproportionately higher incidence found in the younger population <45 years old [2]. BMI is strongly associated with metabolic conditions such as NAFLD and is commonly used as a disease predictor. In correlation with a higher NAFLD prevalence, Caucasians report a higher mean BMI than South Asians [3]. However, studies have shown that South Asians face a higher risk of NAFLD than their Western and Arabian counterparts at the same BMI. In South Asia, 63.5% of NAFLD patients were identified to have NASH, higher than that in South America (27%) [4]. Previously thought to affect mainly Western countries [5], NAFLD prevalence is also rising at an alarming rate in Asia, especially in China [2]. Researchers are still investigating how Asians have an increased risk of weight-related issues at lower BMIs, with explanations such as higher central adiposity, insulin resistance, genetic predisposition, and carbohydrate-rich diets [6]. In the face of each ethnicity’s unique factors contributing to NAFLD development, it is important to dwell deeper into how these heterogeneous factors may affect therapeutic development in today’s translational NAFLD scene.

### 1.2. Pathogenesis of NAFLD

There are various theories surrounding NAFLD pathogenesis, with the most prevalent two theories being the “two hits hypothesis” and “multiple hits hypothesis” [7]. The “two hits hypothesis,” now obsolete, describes the first hit (high-fat diets, obesity, sedentary lifestyle, and insulin resistance) to cause hepatic steatosis, predisposing the liver to a second hit that triggers hepatic inflammation, ballooning, and fibrosis. On the other hand, the “multiple hits hypothesis” is now regarded as the more accurate theory delineating the mechanism of NAFLD progression and consists of multiple factors working in tandem to develop NAFLD.

*NAFL.* NAFLD consists of nonalcoholic fatty liver (NAFL) and nonalcoholic steatohepatitis (NASH). NAFL is characterized by hepatic steatosis without hepatocyte inflammation or ballooning, while NASH is characterized by hepatic steatosis with hepatocyte inflammation or ballooning. In NAFL, fat accumulates in the liver in the absence of excessive alcohol consumption, infections, and autoimmune diseases [2]. The largest contributor to hepatic fat is adipose-derived circulating free fatty acids (FFAs) (59%), which are stored as triglycerides (TGs), the predominant fat found in the livers of NAFLD patients. Hypertriglyceridemia is commonly found in patients with poorly controlled T2DM, obesity, and sedentary lifestyles, heavily predisposing them to NAFL development due to lipotoxicity [8]. Insulin resistance is a key factor in intrahepatic fat accumulation, increasing FFA levels for hepatic accumulation and intrahepatic *de novo* lipogenesis and promoting adipose tissue dysfunction, which reduces adiponectin secretion while increasing proinflammatory cytokine release [2]. The second largest contributor of hepatic fat is *de novo* lipogenesis from non-lipid precursors, mainly glucose and fructose. Diets high in sugar combined with sedentary lifestyles put patients at high risk [9]. Interestingly, TG accumulation may not be as hepatotoxic as we think and could be a defensive mechanism in response to excess FFA levels. A study has shown that triacylglycerol (TAG) accumulation acts to protect the liver from hepatotoxic FFA and does not directly cause liver injury, hence occurring as an epiphenomenon separate from liver injury [10].

First-degree relatives of NAFLD patients have three times the risk of developing NAFLD compared to the general population, pointing to a genetic component in NAFL development [11]. The most common genetic variant identified is PNPLA3 I148M. Loss of function of PNPLA3 reduces TG turnover, leading to TG accumulation in the liver and promoting fibrosis via hepatic stellate cell activation. Reduced expression of PNPLA3 I148M was observed to reduce liver damage. Another common genetic variant is TM6SF2 E167K. Loss of function of TM6SF2 encourages steatosis by preventing the exportation of TG via VLDL. Notably, NAFLD has a high degree of gene-environment interaction, as evidenced by obesity significantly amplifying the risk of NAFLD in genetically predisposed patients.

*NASH.* While it was previously thought that NAFL must precede NASH, studies have shown that NASH itself can be an initial liver lesion. Twenty-five percent of NAFL patients still progress to NASH within three years, with lipotoxicity from excess fats causing hepatocyte inflammation, ballooning, and fibrosis [12]. In NASH, the body enters a proinflammatory state from intrahepatic fat-induced lipotoxicity. High levels of proinflammatory cytokines, such as TNF-α, IL, DAMPs, and PAMPs, are released. Kupffer cells and neutrophils are also activated, exacerbating hepatocyte injury. In NASH patients, increased lipid burden is mirrored by increased mitochondrial activity, which in turn leads to mitochondrial exhaustion. Mitochondria are responsible for the β-oxidation of FFAs, ATP synthesis, and ROS production [13]. Hence, lipotoxicity-induced mitochondrial dysregulation results in enhanced hepatotoxic ROS and increased mitochondrial sensitivity to TNF-α.

A study showed that transplantation of gut microbiota from lean patients increased insulin sensitivity in obese patients [14]. This suggests the presence of “obese microbiota” and “lean microbiota”, which have different capacities in absorbing fats in the gastrointestinal tract. NAFLD patients were also found to have compositional differences and larger volumes of microbiota with a higher prevalence of small intestine bacterial overgrowth (SIBO) (77%) than healthy individuals. SIBO leads to increased intestinal permeability, proinflammatory cytokines, and dysregulated bile acid metabolism. Bile acids activate the farnesoid X receptor (FXR), which is responsible for lipogenesis and VLDL export, linking bile acid metabolism to NAFLD development. However, variables are difficult to control due to different diets, lifestyles, as well as antibiotic and microbiota exposure; hence, the results are not the most conclusive.

NAFLD patients with NASH, fibrosis, or associated T2DM are at high risk of NAFLD progression and must be identified early to prevent complications. Hepatic complications include cirrhosis, leading to hepatocellular carcinoma (>50% mortality), and hepatic emergencies, such as esophageal variceal bleeding (15% mortality) [15]. Patients are also at risk of nonhepatic complications, including cardiovascular diseases (40% mortality), T2DM and chronic kidney disease [16].

*Cirrhosis and hepatocellular carcinoma (HCC).* Approximately 40% of NASH patients develop fibrosis, which is double the fibrosis progression rate compared to NAFL [2]. Patients with NAFLD with fibrosis are at higher risk of adverse outcomes, such as cirrhosis and HCC [17]. Constant inflammation and regeneration of the hepatocyte, along with added stress from dysfunctional fibrosed hepatocytes, predisposes to neoplastic formation in the liver. Even without cirrhosis, 50% of HCC cases are associated with NAFLD [18]. One UK study discovered that cases of HCC secondary to NAFLD increased by 10-fold within the last decade [2].

### 1.3. Risk Factors for NAFLD

In a global study, T2DM and obesity were found in 22.5% and 51% of NAFLD patients, respectively [2]. Furthermore, NAFLD prevalence is almost double that in T2DM and obese patients compared to the general population. The bidirectional relationship between T2DM and NAFLD also means that either condition will lead to the other, which is clearly an important risk factor to address.

While obesity is a risk factor for NAFLD, it is unlikely to be the main driver of fibrosis progression after NAFLD onset. Lean NAFLD patients with BMI <25 are found more commonly in Asia (19%) than in South America (7%). Furthermore, lean NAFLD exhibits worse insulin resistance, dyslipidemia, and hypertension than the general population but has better cardio-metabolic risk than obese NAFLD [2]. A study has shown that lean NAFLD patients have accelerated fibrosis progression over 20 years compared to obese NAFLD patients, but this remains controversial [19]. with both lean and obese NAFLD exhibiting worse insulin resistance than the general population, insulin resistance may be an important driver in NAFLD progression. Other endocrine risk factors for NAFLD include hypogonadism, glucocorticoid excess, hypothyroidism, menopause, and growth hormone deficiency [2].

## 2. Diagnosis and Prognosis of NAFLD

After more than 10 years of active investigations, alternatives to liver biopsy for staging chronic liver diseases have revealed both their strength and weakness. Liver biopsies predict disease progression, while noninvasive markers predict NAFLD diagnosis and NAFLD-related morbidity and mortality. This implies that risk stratification of NAFLD patients can be carried out purely by noninvasive markers, with high-risk patients undergoing recommended biopsies, while low-risk patients continue with noninvasive markers only. Moving forward, using a combination of biomarkers with imaging techniques may yield the most accurate diagnostic capabilities via noninvasive measures.

### 2.1. Noninvasive Methods for NAFLD

Although hepatic steatosis was initially defined as a diagnostic histological feature for NAFLD diagnosis, recent years saw the emergence and clinical adoption of various noninvasive diagnostics through MRI-proton density fat fraction (MRI PDFF), magnetic resonance spectroscopy (MRS), and computed tomography (CT). Early detection of NAFLD is key to effective treatments and better management of the disease. There is an increasing reliance on noninvasive methods to diagnose and stage NAFLD. Not only are these methods more relevant in detecting early-stage asymptomatic NAFLD, but they also have better risk-benefit ratios than invasive methods.

*MRI PDFF.* Hepatic steatosis is heavily associated with chronic liver disease, especially in the earlier stages of NAFLD. In addition to histological changes, steatosis severity has been found to be linked to the development of fibrosis and NASH progression [20]. Currently, MRI-proton density fat fraction (MRI PDFF) is the most accurate noninvasive quantifier of hepatic steatosis reduction in clinical trials. A 2-point improvement in NAS can be identified by MRI-PDFF via a 30% reduction in hepatic fat content [21]. Hence, the drug responses of NAFLD patients with simple steatosis or NASH without fibrosis can be accessed via hepatic fat content measurements by MRI PDFF [22]. MRI PDFF is advantageous, as it performs whole liver mapping to determine hepatic fat content compared to magnetic resonance spectroscopy, which only covers a selected area of the liver. However, MRI-PDFF is user dependent, with variable performance based on technical skills, and cost expensive.

*MRS.* MRS is the mainstay imaging modality for grading hepatic steatosis in clinical trials, as it is not affected by concomitant liver conditions, iron deposition, or fibrosis, unlike other noninvasive methods. It can detect, grade, and quantify hepatic fat with high sensitivity and specificity. However, MRS is also user-dependent and expensive. NAFLD is usually defined as >5% hepatic fat content measured by MRS. This value, however, is almost the same as the absolute hepatic fat reduction cutoff (>5%) and can restrict the reported proportion of patients undergoing hepatic fat reduction, especially for those with hepatic fat slightly above 5%. NASH patients typically also have at least 20% hepatic fat content along with inflammation, which can be used as another surrogate marker of hepatic fat reduction [23]. Hepatic fat reduction as a predictor of histological improvement in NASH is a highly contested topic. According to studies, a decrease in at least 5% absolute liver fat content or 30% relative liver fat content measured by MRI-related methods is required for overall histological improvement [24,25].

*CT.* CT measures hepatic steatosis via radiation attenuation, with higher sensitivity and specificity for more advanced NAFLD [26]. However, ionizing radiation in CT limits its usage in at-risk populations, such as children and pregnant women. The main limitation of imaging techniques such as MRI-PDFF and CT, when compared to biopsies or biomarkers, is the inability to determine if the steatosis is reactive to infectious or inflammatory conditions, such as hepatitis or alcohol-induced or secondary to metabolic syndrome.

*Biomarkers*. AST and ALT are the most closely associated biochemical markers with hepatocellular injury and can be used to predict overall NAFLD severity and mortality. The FLINT trial demonstrated how ALT reduction in at least 17 IU/L is associated with histological improvements. However, the nonspecific nature of these liver enzymes makes it difficult to diagnose NAFLD in asymptomatic patients. For example, despite the almost universal reliance on transaminase levels in real-life practice, liver enzyme levels are not considered useful for diagnosing NASH. Most patients have normal transaminase levels, but this does not rule out histologically advanced disease. Finding biomarkers that can identify patients at high risk of NAFLD and quantifying the severity of the disease will prove very useful in both clinical trials and practice.

*Homeostatic model assessment of insulin resistance (HOMA-IR)*. HOMA-IR is an affordable, noninvasive diagnostic method for NAFLD in T2DM patients. It is calculated using the following formula: [plasma glucose (mg/dL) ∗ plasma insulin (μU/mL)]/405. A study concluded that HOMA-IR is independently associated with NAFLD in T2DM patients, with decent sensitivity (66%) and high specificity (93%) and rivaling imaging techniques [27]. The main limitation of utilizing HOMA-IR lies in only evaluating the patient’s insulin sensitivity [28]. In individuals with nondiabetic glucose intolerance, fasting glucose and insulin concentrations are highly correlated positively. When a patient’s glucose tolerance deteriorates into a diabetic state, insulin concentrations start to decline and exhibit an inverse relationship to glucose rather than a positive relationship [29]. Under such conditions, HOMA-IR underestimates insulin resistance-associated NAFLD.

### 2.2. Noninvasive Methods for NAFLD with Fibrosis

Fibrosis is a strong predictor of NAFLD patient morbidity and mortality. Given the nonlinear progression of fibrosis, fibrosis must be regularly reassessed over time. However, repeated biopsies pose a significant risk to the patient, and the patient will also be unwilling to return for follow-up. Noninvasive measures that can perform at the same level, if not better than liver biopsies, will be key in the risk stratification and management of NAFLD patients.

*FibroScan (transient elastography)*. FibroScan is a form of ultrasound. Ultrasound is used to look for hepatic steatosis, with decent sensitivity and specificity. It produces a low-frequency wave and correlates the wave velocity to liver stiffness, measuring the extent of liver steatosis and fibrosis [30]. Compared to noninvasive biomarkers such as the AST to platelet ratio index (APRI), FibroScan is significantly more accurate in predicting all-cause mortality in NAFLD patients [31]. However, the measurement of liver stiffness in FibroScan may be inaccurate depending on inter-operator differences, abdominal adiposity, or the size of the intercostal space. There is also variability in cutoff liver stiffness measurement values, but no studies to date have specifically addressed this clinical predicament [32]. FibroScan is not as effective in obese individuals, which make up a large proportion of NAFLD patients.

*Biomarkers.* Studies have shown that Fibrosis 4 (FIB-4), NAFLD fibrosis score (NFS), and APRI have limited ability to predict fibrosis progression but are on par or even better than liver biopsies in predicting liver-related morbidity [31]. The FIB-4 index and NFS are both the most accurate in predicting all-cause mortality, while NFS has the best prognostic accuracy for fibrosis progression, especially at advanced stages beyond F3. Other predictors of fibrosis include BAAT score, BARD score, Fibrometer, HAIR algorithm, and extracellular matrix turnover markers such as the European Liver Fibrosis test (ELF), hyaluronic acid, P3NP, and T1MP1 [33]. Currently, the best fibrosis predictors are hyaluronic acid, P3NP, and T1MPI for detecting F1 and F3, and fibrometer for F2.

### 2.3. Invasive Methods for NASH

*Liver biopsy.* Liver histology via biopsy remains the “imperfect” gold standard diagnostic tool for NAFLD. Histological staging scores include NAS and SAF (steatosis, activity, fibrosis), which are commonly used as primary and secondary outcomes in clinical trials. NAS is a measure of disease progression and is not the same as the clinical diagnosis of definite steatohepatitis. Each scoring has its own criteria (Table 1 and Table 2). However, there are many downsides to an overreliance on biopsies, not only in clinical research but also in clinical practice. The invasive procedure has a risk of rare but life-threatening complications and is not ideal for monitoring patients over time. It is a costly procedure that may require the additional cost and time of an interventional radiologist. Patients are also concerned about the invasive nature of the biopsy and the potential for pain, discomfort, and complications. Thus, the number of patients with NASH is underdiagnosed. Finally, a biopsy analyses 1/50,000 of the liver, and interpretation may differ between pathologists; therefore, serial biopsies may provide inconsistent findings.

## 3. Current Management of NAFLD

NAFLD management encompasses a multidisciplinary approach, given the high hepatic and nonhepatic complications suffered by NAFLD patients. Despite our vast knowledge of its etiology, the first-line management of NAFLD is lifestyle modification, which is often mandatory interventions in most patients [36]. It encompasses dietary changes, weight loss exercise programs, and smoking cessation that target both hepatic and nonhepatic areas (Table 3). with shifts in epidemiology toward the younger population and the rising global burden of NAFLD-related complications, the search for effective NAFLD pharmacological treatments to supplement current treatment regimens has never been so important. By learning from both successes and failures of past clinical trials, this process can be expedited.

The primary mode of intervention is lifestyle modification for both NAFL and NASH. In the case of ineffective treatment by lifestyle modification, NASH patients diagnosed via biopsy have extended therapeutic options in pharmacotherapy. An obese patient may opt for bariatric surgery, while liver transplantation is only considered for end-stage complications such as liver cirrhosis and hepatocellular carcinoma.

While nonsurgical weight loss programs are often effective in improving the liver histologically, long-term compliance is difficult to sustain in target patient groups. Those who find it difficult to achieve sustained weight loss may opt for bariatric surgery [37]. Bariatric surgery significantly reduces stomach volume and decreases nutrient absorption, which improves metabolic pathways, such as lipid, glucose, and inflammatory activities [38]. Surgery is generally safe and beneficial for higher-morbidity patients, even those with well-compensated cirrhosis [37]. However, potential side effects and postoperative complications should be carefully considered [39]. In the case of severe liver complications, liver transplantation is also considered an intervention. However, the risk of recurring and de novo NAFLD is common even after liver transplantation [40].

To date, there are no approved drugs by the FDA to reduce this risk. Nevertheless, the international guidelines have included the use of pioglitazone, a peroxisome proliferator-activated receptor (PPAR) agonist, and/or vitamin E to specifically treat NASH patients (Table 3 and Table 4). This is because patients with fibrotic development usually have a poorer clinical prognosis, and lifestyle modifications may be insufficient in liver histological improvement [41]. The recommended therapy duration ranges from 6 to 24 months, and it is advised to halt pharmacotherapy treatment if serum AST levels do not decrease (Table 4). Various novel hepato-centric drugs have also entered clinical trials to ascertain their feasibility in NAFLD treatment. However, there is no approved pharmacotherapy for NAFL yet, which is a potentially important juncture in NAFLD management. Adopting a prophylactic approach against progression to NASH reduces the risk of complications and increases the chances of full reversal in NAFL compared to NASH, significantly improving the morbidity and mortality of NAFLD patients.

Furthermore, the management of underlying conditions such as T2DM, hyperlipidemia, and metabolic syndrome commonly found in NAFLD patients necessitates the use of pharmacological therapy. Considering the close relationships between such underlying conditions and the pathophysiology of NAFLD, repurposing these drugs to simultaneously treat NAFLD is also a promising avenue to explore.

Taking the next step forward in NAFLD management is a foreboding task wrapped with much uncertainty. However, the demand for NAFLD pharmacological therapy is high and certain. with the perspectives of the challenges and lessons learned from reviewing prominent NAFLD drug trials, this paper aims to provide a stepping stone for future experimental drug trials in their endeavor for more efficacious and safer NAFLD management.

## 4. Methods

**Figure 1:**
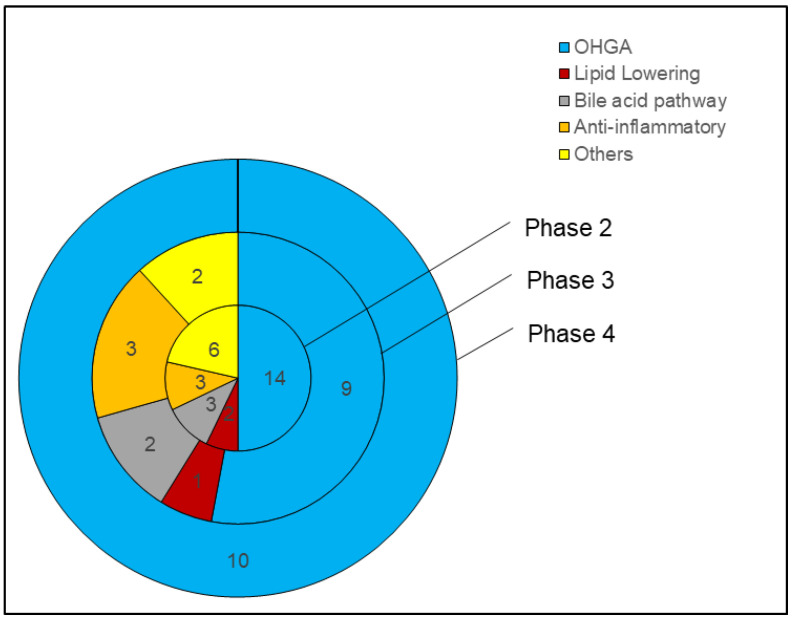
The landscape of NAFLD clinical trials is evaluated in this study.

In this review, we interrogated ClinicalTrials.gov, a registry of clinical trials run by the U.S. National Library of Medicine. The trials presented in this review are as of 24 August 2022. Most trials target overweight white individuals in America and Europe, while a few were carried out in the Middle East and Asia. Nearly half of patients have T2DM or are taking diabetic medication, with 30% taking statins. In search for new interventions to treat NAFLD, various drugs have been studied, including anti-inflammatory, anti-diabetic, lipid-lowering, and bile pathway drugs, in recent years. We collected information on the trial identifier; trial title or acronyms; duration; the number of patients involved and their diagnosis; trial results, and side effects. All trials presented are interventional studies with the primary purpose of treating NAFLD. These eligible trials are required to have published results to be evaluated in this review. We did not include trials that involved diseases such as plaque psoriasis, osteoarthritis, vitamin D deficiencies, hepatitis B, polycystic ovary syndrome, or sexually transmitted diseases such as HIV. Trials involving only dietary or lifestyle changes as a mode of intervention were also excluded. Together, we categorized the studies into 5 different classes: oral hypoglycemic drugs (OHGA), lipid-lowering drugs, bile pathway drugs, anti-inflammatory drugs, and others (e.g., nutraceuticals and microbiome). Figure 1 represents the broad landscape of different interventions studied in NAFLD clinical trials in this paper. OHGA was thoroughly studied, comprising ~50% of phase 2 and 3 trials, while it encompassed all phase 4 trials. Given its variety, nutraceuticals and antibiotics comprise the second largest proportion under the “others” category. Bile acid pathway drugs and anti-inflammatory drug trials comprise a similar proportion, while lipid-lowering drug trials make up the smallest category.

Furthermore, OHGAs included 10 drugs, with 7 at or having completed phase 3 trials and 1 having a terminated phase 3 trial (Table 5). Lipid-lowering drugs included 4 drugs, with 1 in phase 3 trials. Bile pathway drugs included 3 drugs, with 1 in phase 3 trials. Anti-inflammatory drugs included 3 drugs, with 2 having terminated phase 3 trials. There were 2 other drugs at or having completed phase 3 trials. Trials also included 4 combination drugs and 11 repurposed drugs. Beyond pharmaceuticals, 5 nutraceuticals and gut microbiota were also considered for NAFLD management.

Pioglitazone and vitamin E remain the most trialed candidates for NAFLD management. Promising phase 3 drugs such as lanifibranor, dapagliflozin, empagliflozin, aramchol, and resmetirom are garnering more support, while phase 2 drugs such as oltipraz and NGM282 show great potential.

## 5. Oral Hypoglycemic Drugs (OHGA) for NAFLD

### 5.1. PPAR Agonist

*Pioglitazone.* PPARs are nuclear receptors that function as transcription factors involved in energy production and glucose and lipid metabolism. They are comprised of three isoforms, PPARα, PPARβ/δ, and PPARγ, each having their individual roles and tissue distributions. PPARα is most prominently expressed in the liver. It controls hepatic fat levels by influencing fatty acid transport and β-oxidation [66]. Plasma lipids are also reduced by lowering triglycerides and increasing high-density lipoproteins (HDL). In severe NASH, hepatic PPARα is reduced but can recover with the reversal of NASH [67]. PPARβ controls lipid metabolism in the liver and peripheral tissues by regulating fatty acid transport and β-oxidation, increasing HDL, and improving insulin sensitivity while inhibiting hepatic gluconeogenesis [68]. However, PPARγ activation enhances insulin sensitivity and glucose metabolism in adipocytes [69].

**Figure 2:**
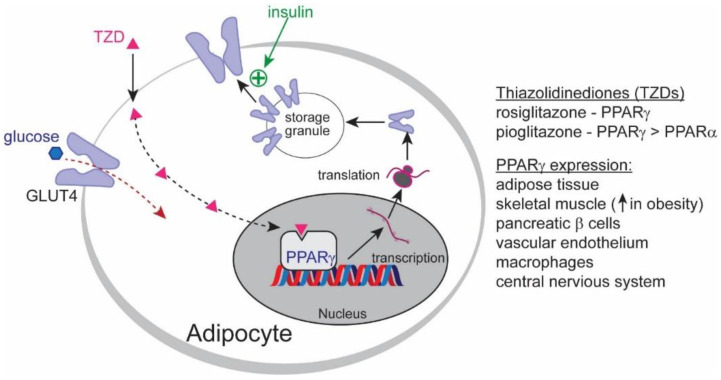
Mechanism of action of pioglitazones. Pioglitazone directly binds and activates PPARγ in the nucleus, increasing the expression of GLUT4 proteins embedded in the cell membranes of storage granules. Upon signaling by insulin, a fusion of storage granules results in the increase in GLUT4 transporters in the cells, increasing the uptake of glucose into cells—thereby increasing insulin sensitivity and glucose tolerance.

Numerous studies have evaluated the efficacy of PPAR agonists, especially pioglitazone, as a treatment for NAFLD. Pioglitazone is a PPARγ agonist used in diabetic patients and repurposed for NAFLD clinical trials. It is an oral hypoglycemic drug that increases insulin sensitivity by stimulating adipocyte differentiation and increasing adiponectin expression. This elicits insulin-sensitizing, anti-inflammatory, and anti-atherogenic properties [69] (Figure 2).

In the UTHSCSA NASH phase 4 trial (NCT00994682) [42], the long-term effects and safety of pioglitazone on hepatic histology in NASH patients were investigated. Pioglitazone showed significant histological improvement in the overall NAS compared to the placebo. Imaging and biomarker improvements were also observed, with hepatic fat content measured via MRS. The number of patients recovering from impaired fasting glucose (IFG)/impaired glucose tolerance (IGT) to normal glucose tolerance (NGT) was significantly higher in pioglitazone than in the placebo, suggesting pioglitazone’s additional role in reducing metabolic risk factors for NAFLD. However, ALT, AST, and adiponectin levels failed to significantly reduce further from 18 months to 36 months, with the placebo decreasing to almost the same level at 36 months. This may indicate a decrease in pioglitazone’s effects after 18 months. Investigating the subsequent effect on histological changes after 18 months could be important in minimizing the duration of pioglitazone administration, potentially balancing pioglitazone’s benefits and side effects. Limitations to study include requirements for a larger, multifactor trial spanning more than 3 years.

In another phase 4 trial (NCT01002547) [43], the long-term effects and safety of pioglitazone and vitamin E on hepatic histology in NASH patients with T2DM were studied. The pioglitazone-vitamin E combination showed significant histological improvement in overall NAS and NASH resolution. Imaging improvements were also observed. However, biomarker improvements in ALT and AST were minimal compared to the placebo. Total cholesterol also increased, with a higher rise in HDL but a lower reduction in LDL compared to the placebo. These changes can be attributed to vitamin E, which demonstrated the highest increase in total cholesterol and triglycerides and the lowest reduction in LDL. This study is limited by a lack of comparison with pioglitazone isotherapy.

The pioglitazone-vitamin E combination demonstrated a higher percentage of patients improving by at least 1 grade in each individual component of NAS, including fibrosis, compared to pioglitazone by >10%. The mean histological scores, number of overall NAS improvements, and NASH resolutions remained similar between both groups. This raises the possibility of a synergistic effect between vitamin E and pioglitazone, having achieved the same results, if not better, within 18 months instead of 36 months. However, the rise in cholesterol and minimized reduction in LDL can be concerning for many NAFLD patients with overweight or hyperlipidemia issues.

In the PIVENS phase 3 trial (NCT00063622) [44], the effects of pioglitazone and vitamin E on hepatic histology in non-T2DM NASH patients were investigated and compared. Pioglitazone showed significant histological improvement in NASH resolution (47%) compared to placebo (20%) but not in overall NAS. There were significant improvements in steatosis and inflammation but not in ballooning and fibrosis. This could be due to a higher proportion of patients classified as not having ballooning at baseline in the pioglitazone group compared to the placebo group, invariably resulting in insignificant overall NAS improvement despite significant NASH resolution. However, the NASH resolution criteria were less strict in this trial compared to the other phase 4 trials, as it did not consider the absence of worsening fibrosis and did not require inflammation and ballooning reduction to 0.

In both T2DM and non-T2DM NASH patients, pioglitazone treatment achieved the endpoint of significant NASH resolution, with T2DM NASH patients reported having significant overall NAS improvement compared to non-T2DM NASH patients. The potential benefit of pioglitazone is hinted at in NAFLD management for patients with impaired insulin sensitization, such as in T2DM patients and South Asians, but cannot be confirmed with current data.

Another reason may also lie in pioglitazone group patients having fewer patients with ballooning compared to placebo. This could have underpowered the overall NAS improvement results, as a reduction in ballooning is part of the criteria.

Despite pioglitazone being a part of international guidelines and demonstrating histological improvement in NAFLD patients with T2DM, it has not been officially approved by the FDA. This is possibly due to long-term PPARγ-driven side effects such as peripheral edema, weight gain, and osteoporosis [70,71]. However, pioglitazone is approved by the FDA as an anti-diabetic drug. It is likely that the indications and lower dosage of pioglitazone administered to T2DM patients make it viable. Pioglitazone is never used as a monotherapy and is only considered when dual therapy with insulin and metformin or sulfonylurea inadequately controls T2DM [72]. Hence, the dosage is generally lower (15–45 mg) than trial dosages (45 mg/day), minimizing adverse effect severity. T2DM patients with uncontrollable blood glucose levels are also likely to experience symptoms worse than the risks of taking pioglitazone. Most NAFLD patients are asymptomatic, making adverse effect tolerance very low.

Obtaining approval for pioglitazone will remain a difficult task unless adverse effects are addressed by adverse effect mitigation. Prophylaxis with low-dosage diuretics can prevent peripheral edema, following a weight loss regime can regulate weight gain issues, and calcium-rich diets with annual DEXA scans can monitor for osteoporotic changes. with patient safety at the forefront, other strategies to monitor for side effects are discussed in Section 11.2.

*MSDC-0602K.* First-generation PPARγ agonists such as pioglitazone are often associated with side effects such as peripheral edema, weight gain, and osteoporosis after high-dose and long-term use [73]. To overcome these side effects, MSDC-0602K was developed. MSDC-0602K is a novel thiazolidinedione mitochondrial pyruvate carrier (MPC) inhibitor that minimizes direct binding to PPARγ. It mirrors the effects of first-generation insulin sensitizers by inhibiting hepatocyte MPC [72], resulting in reduced gluconeogenesis as well as controlled hypoglycemia episodes after a prolonged fast without affecting other hepatic fasting responses [74]. A study [75] with diet-induced obesogenic *db*/*db* mouse models reported that MSDC-0602K reduced insulin resistance, lipogenesis, and gluconeogenesis and increased lipid oxidation in the liver, similar to pioglitazone. These effects were even maintained in hepatocytes without PPARγ, indicating that MPC modulation can substitute for PPARγ inhibition in improving glucose and lipid metabolism. However, mice with diet-induced obesity were unable to fully replicate NASH pathology in humans, and no postmortem mouse liver biopsies were carried out in this study to determine histological benefit.

In the EMMINENCE phase 2 trial (NCT02784444) [47], the efficacy of MSDC-0602K on NASH patients was studied. MSDC-0602K did not show significant histological improvements in overall NAS or NASH resolution. However, there were significant biomarker improvements in HbA1c, fasting glucose, insulin sensitivity, ALT, and AST in the 250 mg and 125 mg MSDC-0602K groups. This suggests that targeting PPARγ plays a larger role in improving hepatic histology while targeting MPC plays a larger role in systemic metabolic changes. Furthermore, another key finding is the improved tolerability in patients treated with MSDC-0602K compared with other PPARγ agonists, e.g., pioglitazone, despite high doses, reducing concerns of overdose-dependent side effects in PPARγ agonists. An ongoing phase 3 study (NCT03970031) [48] on 1800 NAFLD patients with T2DM will evaluate the histological benefits of MSDC-0602K.

*Lanifibranor.* Lanifibranor is a novel pan-PPAR agonist designed by Inventiva Pharma that targets all three PPAR isoforms: PPARα, PPARδ, and PPARγ. Preclinical studies demonstrated anti-inflammatory and antifibrotic effects with higher efficacy than other PPAR agonists, as well as lipid and glucose metabolism regulation [76].

The NATIVE phase 2b trial (NCT03008070) [49] was carried out to determine the efficacy of lanifranor in noncirrhotic NASH patients. Lanifibranor showed significant histological improvement in overall NAS and NASH resolution. Interestingly, the number of patients with NASH resolution and fibrosis improvement of at least 1 stage was significantly higher in the 1200 mg (31.3%) and 800 mg (20.5%) lanifibranor groups than in the placebo (7.4%) group. Comparing this to data on NASH resolution without fibrosis worsening (44.6%, 32.5%, 18.5%) and fibrosis improvement of at least 1 stage without NASH worsening (42.2%, 27.7%, 23.5%), lanifibranor’s antifibrotic effect can be said to have significantly impacted the number of NASH resolution (73.5%, 74.0%, 40.0%). This finding potentially suggests fibrosis improvement is a key factor to more effective improvement in inflammation and ballooning. Significant biomarker improvements, including HbA1c, fasting plasma glucose, HOMA-IR, and adiponectin, were also observed. Raised adiponectin mirrors the results in pioglitazone trials, suggesting similar effects on encouraging lipid metabolism in adipocytes.

Notably, a breakthrough therapy has been granted by the FDA to lanifibranor based on Inventiva’s phase 2b data [77]. A phase 3 study (NCT04849728) [50] investigated the effects of lanibranor in NASH patients with F2–3 compared to placebo on histological improvement on a larger scale and whether there is any significance in delaying NASH disease progression to cirrhosis, liver-related outcome events, and all-cause death.

The side effects of lanifibranor include gastrointestinal disorders, peripheral edema, and weight gain [78], occurring more frequently than placebo. The dropout rate was, however, minimal at 5%. This study sees an opportunity to investigate whether PPARγ-associated side effects, as seen in pioglitazones, are affected by a pan-PPAR mechanism of action, as seen in lanifibranor.

*Saroglitazar.* Saroglitazar is a dual PPARα/γ agonist designed to have a stronger PPARα effect and a weaker PPARγ effect. This promotes lipid and glucose metabolism and insulin sensitization while reducing the severity of PPARγ-related adverse effects. In India, saroglitazar is approved as an anti-diabetic medication for T2DM patients with dyslipidemia unresponsive to statins. In the EVIDENCES IV phase 2 trial (NCT03061721) [51], the efficacy of saroglitazar magnesium in US or biopsy-proven NAFLD patients was investigated. The primary outcome was met in all saroglitazar groups, with significant improvement in ALT and AST compared to placebo. ALT reduction in at least 17 U/L occurred in almost all patients in the saroglitazar groups. Significant imaging improvement in hepatic fat content measured by MRI-PDFF was also observed in 4 mg saroglitazar compared to placebo. Other significant improvements in biomarkers such as ELF, APRI, triglycerides, VLDL, and HOMA-IR were also observed compared to placebo, reducing the risk of cardiovascular complications and improving dyslipidemia in NAFLD patients. Saroglitazar is well tolerated with minimal side effects and is most commonly gastrointestinal. The weaker PPARγ mechanism exhibited a moderate insulin-sensitizing effect while suppressing associated adverse effects such as peripheral edema.

Another phase 2 trial [52] was also carried out to determine saroglitazar’s efficacy in biopsy-proven NASH patients. The primary outcome of overall NAS improvement was not met, as 4 mg saroglitazar was numerically but not significantly higher than placebo. Ballooning was significantly improved in the 2 mg and 4 mg saroglitazar groups compared to baseline, and NASH resolution without fibrosis worsening was significantly higher in both saroglitazar groups compared to placebo. Notably, lobular inflammation experienced greater improvement in the placebo group than in both saroglitazar groups. Saroglitazar also demonstrated a dose-dependent reduction in LDL levels. The lack of a significant primary outcome is likely due to the small sample size of 16 patients.

Additional phase 2 (NCT05011305) [46] and 3 (NCT04193982) [53] trials have been completed but have yet to publish their results on ClinicalTrials.gov. India approved the use of saroglitazar as a NASH treatment in March 2020, providing opportunities for further clinical testing with a larger population sample.

*Elafibranor (TERMINATED).* Elafibranor is a dual PPAR α/β agonist. This drug is one of the lead pipeline drugs of GENFIT to treat NASH [79]. In an animal trial with diet-induced obese mouse models with NASH, elafibranor showed significant histological improvements in steatosis, inflammation, and fibrosis [80]. Such as obeticholic acid, GENFIT’s elafibranor is one of the more promising drugs for PBC treatment (ELATIVE, NCT04526665) [81], having been granted the Breakthrough Therapy Designation by FDA and was recruiting patients for their phase 3 trial.

Although elafibranor had previous success in multiple clinical trials of various diseases, it was not as consistent in NASH. In a phase 2 trial (NCT01694849) by GENFIT [54] to determine the efficacy of elafibranor in noncirrhotic NASH patients, 120 mg elafibranor demonstrated significant histological improvement in overall NAS and NASH resolution without fibrosis worsening compared to placebo. Significant biomarker improvements, including ALT, AST, HbA1c, triglycerides, LDL cholesterol, and HOMA-IR, were also observed compared to the placebo. However, the subsequent RESOLVE-IT (NCT02704403) [55] phase 3 was terminated. RESOLVE-IT investigated the histological effects of elafibranor in NASH patients with F1–3, as well as all-cause mortality and liver-related outcomes. During the interim analysis, elafibranor did not meet the predefined primary outcome of significant NASH resolution without fibrosis worsening, with a response rate of 19.2% in the elafibranor group compared to 14.7% in the placebo group [82]. No significant histological improvements were noted in fibrosis improvement of at least 1 stage in the elafibranor group (24.5%) compared to placebo (22.4%). Secondary outcomes of metabolic parameters such as HOMA-IR and cholesterol levels were also not met. The termination was not due to safety concerns but from the conclusion that participants would derive limited benefit from continuing the trial. Although the failure of elafibranor to attain its primary and secondary outcomes compound;in GENFIT’s termination of this compound, the safety of the drug was consistent with previous Phase 2 trials of no adverse effects.

**Figure 3:**
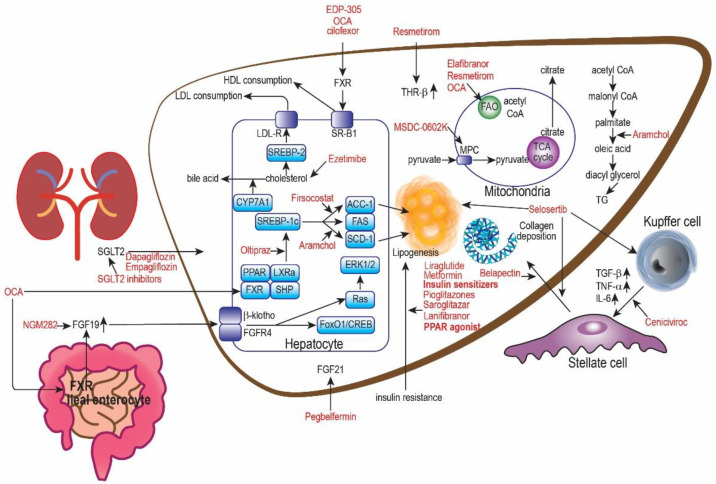
Mechanism of action for various NAFLD pharmacological treatments. Adapted from [90].

*Dapagliflozin.* Dapagliflozin and empagliflozin are sodium-glucose cotransporter 2 (SGLT-2) inhibitors approved by the FDA as oral hypoglycemic drugs in T2DM patients [83]. They inhibit SGLT-2, which is increased in the proximal tubules of kidneys, inhibiting 90% of glucose reabsorption and causing glucosuria [84] (Figure 3). Studies have found that reducing the intracellular glucose concentration triggers a ketogenic effect that shifts glucose metabolism to fatty acid metabolism, reducing triglyceride levels and hepatic steatosis. However, it is still under debate whether this is directly due to SGLT-2 inhibition [85,86,87,88,89]. Nevertheless, SGLT-2 inhibitors are known for weight loss, visceral adipose reduction, improved glucose control, and cardioprotection pointing toward the therapeutic potential for NAFLD [56].

A phase 2 trial [56] investigated the effects of dapagliflozin on hepatic fat levels in NAFLD patients with T2DM. Dapagliflozin had significant imaging improvement in hepatic fat content compared to the placebo, measured via non-contrast CT scan. Significant biomarker improvements in ALT and HbA1c were also observed. Another phase 2 trial [57] investigated the effects of dapagliflozin on sDPP4 levels in NAFLD patients with T2DM. sDPP4 is secreted by hepatocytes, inducing insulin resistance and adipocyte inflammation. Dapagliflozin had significant biomarker improvements in AST, ALT, and HOMA-IR. The study also reported a positive relationship between liver enzymes and sDDP4, suggesting sDDP4 reduction as another potential pathway for SGLT-2 inhibitors to manage NAFLD [57].

This paper is encouraged by dapagliflozin’s positive biomarker results and would recommend following the currently recruiting DEAN phase 3 trial (NCT03723252), which will investigate the histological efficacy and safety of dapagliflozin in NASH patients [58].

*Empagliflozin.* Empagliflozin works along the same pathway as dapagliflozin. Often compared together, empagliflozin has the highest specificity to SGLT2 inhibition to reduce HbA1c more significantly and control fasting glucose, according to a study [91].

In a phase 3 trial (IRCT20190122042450N1) [59], the effects of empagliflozin on liver steatosis and fibrosis in NAFLD patients without T2DM were studied. Imaging modalities such as transient elastography were used to measure the controlled attenuation parameter (CAP) and liver stiffness measurements (LSM) as indicators of hepatic steatosis and fibrosis, respectively. Empagliflozin showed a significant difference in LSM reduction compared to the placebo. There was a significant difference in CAP reduction only in the significant steatosis baseline group when compared to the placebo. There were also significant biomarkers, including AST, ALT, and fasting insulin levels. However, the NAFLD fibrosis score (calculated based on age, BMI, diabetes presence, platelets, albumin, and AST/ALT ratio) and FIB-4 index did not change in either group. Truncal fat mass also increased in empagliflozin, but not significantly compared to placebo. Side effects include mild fungal genitourinary infections. Generally, CAP is less accurate than MRI-PDFF for liver fat estimation, but reliability can increase with stricter thresholds. Significant imaging and biomarker improvements suggest empagliflozin’s potential as a treatment for NAFLD patients without T2DM. However, this will require future trials to place greater emphasis on histological and MRI-PDFF results for comparison with other NAFLD drug trials.

In the E-LIFT phase 2 trial (NCT02686476) [60], the effects of empagliflozin on liver fat content in NAFLD patients with T2DM were studied. Empagliflozin showed significant imaging reductions in liver fat as measured by MRI-PDFF compared to placebo. There was also a significant reduction in ALT but not AST levels. Weight loss and reductions in fasting glucose, HbA1c, and total cholesterol also occurred but were not significantly different from the placebo. Interestingly, serum creatinine increased significantly in the empagliflozin group. This can indicate exacerbation of renal damage already occurring in T2DM. However, the few significant adverse events that occurred did not report any serum electrolyte changes or edema.

Furthermore, novel SGLT2 inhibition-mediated mechanisms have been found to support the targeting of SGLT-2 in managing NAFLD. A study on NAFLD mice [92] with T2DM showed that empagliflozin significantly attenuated liver injury by enhancing the AMPK/mTOR signaling pathway to enhance macrophage autophagy to prevent hepatic injury and inhibiting IL-17/23 for anti-inflammatory function. Clinically, SGLT2 inhibitors are generally well tolerated by patients. Side effects included genitourinary infections (5%), euglycemic diabetic ketoacidosis, and postural hypotension. However, the risk of side effects can be minimized via careful monitoring of one’s genital hygiene, capillary blood glucose levels, meal timings, and sufficient hydration [2]. Currently, future clinical trials studying the effect of empagliflozin on liver fat include two phases 4 clinical trials (NCT03646292; NCT04642261) [45], which are now recruiting.

While there are few to no reported histological results surrounding SGLT2 inhibitors in NAFLD drug trials, positive imaging and biomarker results have been achieved thus far. Ongoing phase 3 and phase 4 trials will provide more evidence of the efficacy of SGLT2 inhibitors in NAFLD management.

### 5.2. GLP-1 Analog

*Semaglutide.* Semaglutide is a glucagon-like peptide 1 (GLP-1) analog that acts on appetite centers in the hypothalamus to enhance satiety and reduce food cravings, promoting weight loss in patients with obesity via caloric deficit [93]. Previous phase 3 trials (NCT02054897, NCT02692716) [94,95] on T2DM patients have demonstrated semaglutide’s ability to improve liver biomarkers, reduce weight, control HbA1c and protect against cardiovascular risk factors. This leads to weight loss, better glycemic control, fewer hypoglycemic events, and cardiovascular protection [96]. Normally, semaglutide is administered as a weekly subcutaneous injection for patients with T2DM and obesity. While there is no approval of semaglutide for the treatment of NAFLD today, Novo Nordisk has been studying its effects on NASH patients.

In its phase 2 trial (NCT02970942) [61], the effect of semaglutide on NASH patients was studied. Semaglutide showed histological improvement in overall NAS and NASH resolution compared to placebo. NASH resolution without fibrosis worsening was also significantly higher in semalgutide than in placebo. However, there was no significant improvement in the fibrosis stage in semaglutide compared to placebo. This was unexpected, considering that NASH resolution was associated with fibrosis regression. This could be attributed to an insufficient length of the trial for significant fibrosis improvement. Semaglutide also preferentially reduced ballooning and inflammation over fibrosis. Biomarker improvement was also observed. Weight loss of >5% was significant in 0.4 mg (76.8%) compared to placebo (16.3%). However, no significant reduction in LDL or HDL occurred in any of the semaglutide groups. Nonetheless, weight loss is key in NAFLD management. Side effects include mild gastrointestinal disorders in the first 20 weeks of administration. This puts semaglutide as the most promising GLP-1 analog for T2DM patients with NASH. Looking forward, Novo Nordisk is currently recruiting 1200 NASH patients in its 5-year-long phase 3 study (NCT04822181) [97], which is estimated to be completed by May 2028. Novo Nordisk’s semaglutide was the first ever orally administered pill version of a GLP-1 agonist and received FDA approval in 2019 for T2DM treatment [98]. It is a once-weekly drug at a dose of 2.4 mg for weight loss in adults with a BMI of 27 kg/m^2^ or greater in 2021—a first since 2014 [97]. This raises questions about any changes to the efficacy and safety profile of semaglutide.

*Liraglutide (phase 2).* Liraglutide is a shorter-acting GLP-1 analog that acts on GLP-1 receptors present in hepatocytes [99]. It has a shorter half-life of 13 h compared to 160 h in semaglutide, hence requiring daily subcutaneous injections [100].

In the LEAN phase 2 trial (NCT01237119) [62], the effects of liraglutide on NASH patients were studied. Liraglutide showed significant histological improvement in NASH resolution compared to placebo but not in overall NAS. The number of patients with steatosis and ballooning reduction was significantly higher in the liraglutide group than in the placebo group but not in the inflammation and fibrosis groups. This is despite significantly fewer patients experiencing fibrosis worsening in the liraglutide group. The only biomarker improvement of significance was GGT. As the standalone liraglutide study investigating NASH histological improvements, this paper recommends future studies of larger sample sizes with a focus on histological improvement and NASH resolution to compare liraglutide’s efficacy with other NASH drug trials.

Liraglutide is considered safe and tolerable, with side effects being mostly mild to moderate and transient. Gastrointestinal disorders such as nausea, diarrhea, and abdominal pain were the more common side effects experienced in the liraglutide group but did not warrant discontinuation of the drug. Following the results, a longer-term, higher sample size phase 3 study is supported.

### 5.3. AMPK Inhibitor

*Metformin (phase 2).* Metformin is the first-line OHGA pharmacological therapy in most T2DM patients [101]. It works by inhibiting hepatic gluconeogenesis and the effects of glucagon, hence improving insulin sensitivity and reducing hepatic fat accumulation. In an open-label randomized trial [63], nondiabetic biopsy-proven NAFLD patients were administered 2 g/day metformin, 800 IU/day vitamin E or a placebo with a prescriptive diet for 12 months. A liver biopsy of the metformin-treated group revealed significant improvement in the necroinflammation score, fibrosis score, and overall NAS. Biomarkers such as ALT and AST, and HOMA-IR also showed significant improvement compared to vitamin E and placebo.

In another trial [102], NAFLD patients were administered up to 3 g/day of metformin or a placebo for 6 months. While histological improvement occurred, no significant histological differences between the two groups were noted. Significant improvements in biomarkers such as ALT and AST, and HOMA-IR were observed in metformin-treated patients. However, further analysis revealed significant differences in improvement found in only NAFL patients and not NASH patients. This suggests that metformin’s benefits are far more limited in patients who have already progressed to NASH compared to NAFL.

In another trial [65] administered up to 1 g/day of metformin or placebo was in non-diabetic patients for 12 months, with both groups undergoing a weight loss program. While histological improvement occurred, no significant histological or biomarker differences between the two groups were noted. However, this study only had a sample size of 19 patients, which may be too small to be statistically significant.

Metformin is generally well tolerated. Side effects of watching out include mild gastrointestinal complications and more severe but rare lactic acidosis. While metformin is a well-known anti-diabetic drug, a more comprehensive double-blind phase 2 trial with a larger sample size on T2DM patients focusing on histological results could provide the necessary push for phase 3 trials.

### 5.4. Comparing PPAR vs. SGLT-2 vs. GLP-1 vs. AMPK Inhibitor

Pioglitazone, lanifibranor, and GLP-1 agonists achieved overall NAS and NASH resolution without worsening fibrosis. Pioglitazones and lanifibranor had the greatest effect on fibrosis improvement. For pioglitazones, this was most prevalent in T2DM patients. Coadministration with vitamin E also served to amplify pioglitazone’s histological benefits. Pioglitazones and SGLT-2 inhibitors reduced hepatic fat levels on imaging but utilized less accurate imaging modalities, such as non-contrast CT scans in dapagliflozin and elastography in empagliflozin. PPAR agonists, SGLT-2 inhibitors, and semaglutide reduced ALT and AST. Pioglitazone and lanifibranor had the greatest effect on adiponectin. Diabetes control was most prevalent in PPAR agonists and SGLT-2 inhibitors. MSDC and empagliflozin had the greatest effect on cholesterol reduction. Dapagliflozin and semaglutide led to the greatest number of patients with weight loss. These results provide the support that pioglitazone is the most promising NAFLD drug for T2DM patients. Lanifibranor has a similar effect as pioglitazone with milder side effects on NAFLD patients with or without T2DM, so phase 3 trial results are to be anticipated.

## 6. Lipid-Lowering Drugs for NAFLD

### 6.1. NPC1L1 Inhibitor

*Ezetimibe (Phase 2)*. Ezetimibe is commonly used in hyperlipidemia to lower LDL, reducing cardiovascular risk. It works by inhibiting NPC1L1, which is responsible for jejunal enterocyte cholesterol absorption. In a phase 2 trial (UMIN000005250) [103], 32 NASH patients were administered 10 mg/day of ezetimibe or a placebo for 6 months. The primary outcome of significantly reduced serum total cholesterol was achieved in the ezetimibe group. Histological improvements were also achieved, with improved hepatocyte ballooning and fibrosis scores (Table 6). However, there was an increase in HbA1c and hepatic long-chain fatty acids. Long-chain fatty acids have been shown to worsen insulin resistance and increase the risk of T2DM [104]. This was severe enough to discontinue the initial plans for 80 NAFLD patients. In order to minimize these side effects, ezetimibe can be taken with insulin sensitizers such as metformin, and low-fat or medium-chain fat-rich diets can be followed [105].

### 6.2. HMG-CoA Inhibitor

*Statins (Phase 2).* Hydroxy-3-methyl-glutaryl-coenzyme A (HMG-CoA) is the rate-limiting enzyme mediating cholesterol synthesis in the liver [109]. Increased expression of HMG-CoA in NAFLD patients contributes to commonly associated comorbidities such as obesity, hyperlipidemia, and increased cardiovascular mortality. Statins are HMG-CoA reductase inhibitors used to lower LDL and triglycerides and increase HDL. Other effects, such as anti-inflammation and reduction in oxidative stress, have prompted the potential benefit of statins to NAFLD patients (Table 6). Currently, only a small number of studies have been carried out to determine the effectiveness of statins in NAFLD. Some small open-label studies have shown improvements in NAS and liver biomarkers [110,111]. Another small-scale double-blind study on 14 patients with biopsy-proven NASH for 12 months demonstrated no significant histological or biomarker improvements [106]. These studies have been limited by their small size and lack of placebo control and thus have been unable to fully demonstrate the effect of statins in NAFLD patients.

Statins are generally well tolerated. One common side effect is asymptomatic temporarily raised liver enzymes occurring early in statin therapy, which could discourage clinicians from prescribing statins to NAFLD patients. Although raised liver enzymes usually point to hepatotoxicity, various studies have attributed this to preexisting liver conditions and risk factors and not directly caused by statin therapy [106]. In a study of hepatic adverse effects in statins [112], significantly increased liver enzymes were observed in only 1% of 927 patients. Furthermore, another study of T2DM patients with NASH demonstrated lower ALT and AST in statin users than in nonusers after 36 months [113].

### 6.3. SCD Inhibitor

*Aramchol.* Aramchol is a novel stearoyl-CoA desaturase 1 (SCD-1) inhibitor developed as an oral drug by Galmed Pharmaceuticals. A fast-track designation was granted by the FDA for the treatment of NASH in 2014. SCD-1 is responsible for lipogenesis via saturated fatty acid conversion to monounsaturated fatty acids (Figure 3) [15]. A phase 2b study (NCT02279524) [107] was carried out to study the efficacy and safety of aramchol in NASH patients with prediabetes or T2DM. The study found that mean hepatic liver fat was significantly reduced in the 400 mg aramchol group (−3.41%) but not 600 mg (−3.18%) compared to the placebo. NASH resolution without fibrosis was also significant in the 400 mg group compared to the 600 mg group. However, there was a significantly higher percentage of patients who experienced a >5% reduction in liver fat in the 600 mg aramchol group (47%) than in the 300 mg group (36.7%). Biomarkers were significantly improved in both aramchol groups, with AST improving more than ALT. The study also found that aramchol was able to significantly reduce HbA1c in NASH patients compared to a placebo. Further analysis elucidated that aramchol also potentially regulates glucose metabolism, explaining the HbA1c reduction [114] (Table 6). However, the same percentage of patients developed cirrhosis in the 400 mg aramchol group and placebo, which was slightly higher than that in the 600 mg group. No significant change in fibrosis improvement was noted between the aramchol group and the placebo group. This indicates aramchol’s poor effect on fibrosis, which, if allowed to progress, will eventually lead to liver-related complications and mortality regardless of NASH resolution. Perhaps future studies can shed light on the effects of combinations with anti-fibrotic drugs.

Based on the at least 5% absolute liver fat reduction cut-off, the results for aramchol were on a similar magnitude to OCA. Unlike OCA, which had the accelerated approval pathway rejected due to pruritus, aramchol is considered safe and well tolerated with no significant side effects according to the ARRIVE trial (NCT02684591) [108,115] (Table 6). Generally, aramchol is well tolerated in NASH patients but requires further investigation in trials with larger sample sizes.

Moving forward, in the ARMOUR phase 3 trial (NCT04104321) [108], the drug’s safety and efficacy were investigated by comparing a 300 mg Aramchol dose to a placebo. ARMOR is currently on an ongoing recruitment status with the aim of studying 2000 adult participants with NASH of fibrosis stage 2 or 3 and overweight to obese demographics.

### 6.4. LXRα Inhibitor

*Oltipraz (phase 2).* Oltipraz is a novel LXRα inhibitor that acts as an antisteatotic agent. It reduces steatosis by inhibiting lipogenesis and activating lipid oxidation. A phase 2 study (NCT01373554) investigated the efficacy and safety of oltipraz in US-proven NAFLD Asian patients with >20% hepatic fat content [23]. Exclusion criteria included patients who took medications such as insulin, insulin sensitizers, vitamin E, and statins. Oltipraz showed a significant reduction in MRS-measured relative hepatic fat content at 120 mg/day oltipraz (−34.6%) compared to placebo (−0.6%). BMI was also significantly reduced in 120 mg/day oltipraz compared to placebo. However, no significant absolute changes were observed in triglycerides, insulin resistance, or adiponectin, and no significant differences were observed in ALT and AST compared to placebo. However, there was a significant increase in HDL for 60 mg/day of oltipraz, possibly contributing to hepatic fat reduction (Table 6).

The BMI-reducing capabilities of orltipraz are especially striking, as this can address not only the overweight risk factor for most NAFLD patients but can also be combined with other potential NAFLD drugs, such as pioglitazone, that cause weight gain. Notably, this trial was carried out on Asian patients only, which holds more significance in our local Southeast Asian context. However, this makes it difficult to compare this trial with most other trials carried out in Western countries with a higher prevalence of obesity.

Oltipraz is considered well tolerated, with a similar incidence of mild gastrointestinal side effects across both oltipraz groups and placebo. with these results in mind, longer-term studies with larger sample sizes could potentially demonstrate the antisteatotic and antifibrotic effects of oltipraz in NAFLD patients in both Asians and Westerners.

## 7. Bile Pathway Drugs for NAFLD

### FXR Agonists

*Obeticholic Acid (OCA).* Farnesoid-X-receptors (FXRs) is highly expressed in the small intestine and liver and are responsible for bile acid, glucose, and lipid homeostasis [116] (Figure 3). FXR activation in the liver reduces bile acid synthesis by inhibiting cholesterol conversion to bile acids and exhibits anti-inflammatory and antifibrogenic activity [90]. FXR activation in the ileum inhibits the uptake of bile acids by downregulating Na-bile acid transporters. Furthermore, an animal study has shown FXR activation protects diet-induced NASH animal models from hepatic steatosis, inflammation, and fibrosis [117]. The multifaceted mechanism of action highlights FXRs as a promising target in NAFLD management (Figure 3).

Obeticholic acid is a synthetic FXR agonist that is 100-fold more potent than its natural counterpart, chenodeoxycholic acid (CDCA) [118]. Among the drug interventions studied to treat NAFLD, obeticholic acid proved to be the most promising. In a phase 2 trial [119], noncirrhotic NASH patients were administered 25 mg/day OCA or placebo for 18 months. There was a significant histological improvement in steatosis, inflammation, ballooning, and fibrosis in OCA-treated patients compared to placebo-treated patients. Furthermore, biomarkers such as ALT and AST were improved. However, increased total cholesterol and LDL and reduced HDL were also observed compared to the placebo.

In 2016, Intercept Pharmaceuticals Inc.’s OCA was approved for the accelerated study of primary biliary cirrhosis (PBC) treatment in adults [120]. Intercept’s OCA is also one of the few NASH drugs with a breakthrough therapy designation due to the demonstrated promise of their REGENERATE trial interim results [121].

In the REGENERATE phase 3 trial (NCT02548351) [122], the histological effects and liver-related clinical outcomes of OCA on noncirrhotic biopsy-proven NASH patients with F2–3 or F1 with obesity, T2DM or ALT >1.5× the upper limit of normal. The first short-term primary outcome was met with significant histological improvement in fibrosis of at least one or more stages and no worsening of NASH in the 25 mg/day OCA group compared to the placebo group. Significant improvement in fibrosis by at least 1 stage occurred in both the 25 and 10 mg/day OCA groups compared to the placebo group, while significant improvement in fibrosis by at least two stages occurred in the 25 mg/day OCA group only compared to the placebo group.

The second short-term primary outcome of NASH resolution with no worsening of fibrosis was not met. However, there was a significant histological improvement in inflammation and hepatocellular ballooning in the 25 mg/day OCA group compared to the placebo group. This could be due to the recruitment of more advanced NAFLD patients who have higher inflammation and ballooning baseline scores, making it difficult to reduce both scores to 0 and 1 for NASH resolution. Secondary outcomes, such as biomarkers, showed favorable decreases in ALT, AST, and GGT. LDL increased the most for the 25 mg/day OCA group and the least for the placebo group. This rise in LDL is concerning, as it may contribute to already rising cardiovascular risk in NAFLD patients. However, the overall cardiovascular risk of OCA in NAFLD patients is still under question, given OCA-driven weight loss and reduced triglyceride levels.

The results imply that OCA has a stronger effect on fibrosis than inflammation or ballooning. However, post hoc pathologist diagnostic assessment reported NASH resolution in twice as many 25 mg/day OCA patients as in placebo. This assessment is more clinically relevant as the pathologist definition is used to define NASH in clinical practice, and assessment based on NAS parameters can be too rigid for real-life practice. Hence, OCA may have a stronger than reported effect on inflammation and ballooning, which can also be further investigated with more clinically relevant parameters.

Alarmingly, 51% of the 25 mg/day OCA group participants experienced a common adverse effect, pruritus, compared to 28% in the 10 mg/day OCA group and 19% in the placebo group. The prevalence of serious adverse events was similar across all treatments (11–14%) (Table 7). As such, despite the clinically significant histological improvement from the interim study, the FDA rejected the accelerated approval pathway in June 2020 due to concerns about adverse side effects and risks [115]. Nevertheless, the upcoming 7-year-long postinterim study for REGNERATE will evaluate the effect of OCA compared to placebo on long-term safety, liver-related, and mortality outcomes.

*Cilofexor (GS-9674) (Phase 2).* In order to overcome the side effects of systemic FXR agonism by OCA, a novel selective non-bile acid FXR agonist was developed. By avoiding enterohepatic circulation, cilofexor primarily targets intestinal FXR receptors to stimulate fibroblast growth factor 19 (FGF19) release while avoiding systemic side effects such as pruritus, hyperlipidemia, and hepatotoxicity (Figure 3). A proof-of-concept study [125] exhibited reduced hepatic fat and improved liver biomarkers such as ALT and AST in patients with NASH and F2–F3 fibrosis.

A phase 2 trial (ATLAS) (NCT03449446) [123] investigated the safety and effect of cilofexor, selonsertib, and firsocostat on liver fibrosis in biopsy-proven NASH patients with F3–4. Firsocostat inhibits acetyl-CoA carboxylase (ACC), preventing the conversion of acetyl-CoA to malonyl-CoA for de novo lipogenesis [126]. Selosertib inhibits apoptosis signal-regulating kinase, halting the progression of lipotoxic-induced inflammatory cascades to protect the liver [127]. Cilofexor did not meet the primary outcome, as there was no significant one- or more-stage improvement in fibrosis without NASH worsening compared to the placebo. On the other hand, cilofexor-firsocostat (21%) and cilofexor-selonsertib (19%) combinations had a higher proportion of one or more stage improvement in fibrosis without NASH worsening compared to cilofexor isotherapy (12%). Cilofexor-fircostat also showed significant histological improvement in 2 or more points of NAS reduction, steatosis, inflammation, and ballooning reduction compared to placebo. Significant imaging and biomarker improvement was also observed in liver stiffness measured by transient elastography, ALT, AST, bilirubin, cytokeratin-18, and insulin. Cilofexor patients experienced the highest percentage of treatment-emergent adverse events (TEAEs) compared to selonsertib, firsocostat isotherapy, and placebo, with up to 29% of patients reporting pruritus compared to placebo (15%). with the high proportion of cirrhotic (56%) and NAS >5 (83%) patients tested; this trial mostly looked at the effects on very advanced NAFLD patients. While this highlights the anti-fibrotic effects of the cilofexor-firsocostat combination, the level of fibrosis and steatohepatitis may be too high to observe the full effects of cilofexor isotherapy.

In order to investigate the side effects of cilofexor, another phase 2 study administered 100 mg/day, 30 mg/day cilofexor or placebo to 140 biopsy-proven noncirrhotic NASH patients for 24 weeks (NCT02854605) [62]. Cilofexor showed a significant improvement in hepatic fat content measured by MRI-PDFF compared to placebo. Notably, the 100 mg/day cilofexor group improved by −22.7% compared to the 30 mg/day group, which only improved by −1.8%. Significant biomarker improvements in GGT, bile acids, and C4 were also noted in both cilofexor groups compared to placebo. This finding indicates the effect of cilofexor on an earlier form of NASH. The results showed that a higher percentage of patients in the 100 mg cilofexor group (14%) experienced pruritus compared to the other 2 groups. On the other hand, there were no significant differences between the 30 mg cilofexor group (4%) and the placebo (4%). This indicates the dose-dependent nature of side effects caused by cilfoexor, albeit still well tolerated. This paper encourages further studies into the severity of pruritus and its effectiveness in improving NAFLD histology at lower doses compared to OCA. Other novel FXR agonists, such as nidufexor [128], are currently in phase 2 trials (Table 7).

*EDP-305 (Phase 2).* EDP-305 is another novel selective FXR agonist developed to treat NASH. In vivo studies [128] show that EDP-305 regulates FXR target genes and bile acid and lipid metabolism and demonstrates hepatoprotective effects in rodent models of NASH. The recently completed phase 2 study [124] aimed to assess the function and safety of EDP-305 in patients with fibrotic NASH. A total of 134 patients with fibrotic NASH were administered 2.5 mg/day, 1.5 mg/day EDP-305, or placebo for 12 weeks. The results showed a significant reduction in hepatic fat and ALT in the EDP group compared to the placebo group (Table 7).

Common adverse effects included pruritus, gastrointestinal disorders, headache, and dizziness. Pruritus occurred in the 2.5 mg (50.9%), 1 mg (9.1%), and placebo (4.2%) groups, leading to a whopping 20.8% of 2.5 mg discontinuing the study compared to 1.8% in the 1 mg group. The positive results provide support for continued longer-term studies of EDP-305, but the side effects may hinder studies with higher dosages.

## 8. Anti-Inflammatory Drugs for NAFLD

### 8.1. Phosphodiesterase Inhibitors

*Pentoxifylline (PTX) (Phase 2).* PTX is a phosphodiesterase inhibitor used for peripheral vasodilation in intermittent claudication [129]. PTX also exhibits anti-inflammatory activity against TNF-α, which contributes to NAFLD development. In a phase 2 trial (NCT00267670) [130] to determine the effectiveness of PTX in NASH, 26 patients with biopsy-proven NASH were administered 1200 mg/day PTX or placebo for 12 months. PTX showed histological improvement in overall NAS and fibrosis but was insignificant compared to the placebo. Biomarker improvements in AST, ALT, TNF-α, and adiponectin were noted but were also insignificant compared to placebo. Notably, triglyceride levels increased in the PTX group (5.7%) compared to the placebo group (−24.6%), with no change in weight in either group. Interestingly, the expression of collagen 1, a key biomarker of hepatic fibrogenesis, decreased significantly in the PTX group compared to the placebo group. This indicates some potential effect in fibrosis change that PTX may exert in longer-term trials.

Another phase 2 trial (NCT00590161) [131] with 55 T2DM patients with biopsy-confirmed noncirrhotic NASH administered 1200 mg PTX or placebo for 12 months. PTX showed significant histological improvements in the overall NAS of at least 2 points, steatosis score, and lobular inflammation compared with the placebo group. The absolute reduction in ballooning and fibrosis was significantly higher in the PTX group than in the placebo group, but the difference in the proportion of patients was insignificant. Significant biomarker improvements were also observed in ALT compared to placebo but not in TNF-a, HOMA-IR, or adiponectin. The side effects of PTX are mostly mild, particularly headaches and gastrointestinal disorders. The difference in the histological results in both trials could be attributed to the inclusion criteria of cirrhotic patients in the latter trial, which could have undermined the effects of PTX.

In another trial [132], the effects of fenofibrates and the fenofibrate-PTX combination in biopsy or US-proven NAFLD patients were investigated. Both groups showed similar results in reducing total cholesterol, HDL-C, LDL-C, and triglycerides. Fenofibrate-PTX showed significant improvement in TNF-α, insulin, FBG, HOMA-IR, AST, ALT, and liver stiffness measured by FibroScan compared to fenofibrate isotherapy (Table 8). The combination of fenofibrate-PTX promotes a lipid-lowering effect while maintaining its anti-inflammatory ability, highlighting its potential in future drug combinations.

The mechanisms of PTX are highly controversial, with different studies offering new insights. A study [133] on NASH mouse models highlighted significant suppression of hepatic preneoplastic lesion development in PTX-treated mice, hinting at potential therapeutic effects against NASH-related liver tumorigenesis. Other studies on combination therapy with fenofibrates and vitamin E in NASH patients have also shown improved results compared to isotherapy.

**Table 8:** Clinical trials on anti-inflammatory drugs evaluated.

Anti-Inflammatory Drugs							
Drug	Mechanism of Action	Trial (Type and Identifier)	Patient (Type and Number)	Dosage	Duration	Results (Histology, Imaging, Biomarker) ✓ X	Side Effects
**Pentoxifylline (PTX)**	Phosphodiesterase inhibitor	Phase 2 NCT00267670 [130]	Adult biopsy-proven NASH 26	**1200 mg/day** OR Placebo	12 months	Histology: X Imaging: N Biomarker: X	Well tolerated
		Phase 2 NCT00590161 [131]	Adult biopsy-proven noncirrhotic NASH with T2DM 55	**1200 mg/day** OR Placebo	12 months	Histology: X Imaging: N Biomarker: ✓ (ALT)	
		Phase 2 [132]	Adult biopsy or US-proven NAFLD 90	**1200 mg/day with 300 mg/day fenofibrate** OR 300 mg/day fenofibrate	24 weeks	Histology: N Imaging: ✓ (via Fibrosacan) Biomarker: ✓ (TNF-α, insulin, FBG, HOMA-IR, AST, ALT)	
**Selosertib**	ASK-1 inhibitor	Phase 3 NCT03053050 (STELLAR-3) [134] **TERMINATED**	Adult NASH with F3 808	**18 mg/day** OR **6 mg/day** OR Placebo	48, 240 weeks	Histology: X (18 mg/day and 6 mg/day did worse than placebo; Did not meet primary endpoint during interim analysis at 48 weeks, hence terminated) Imaging: N Biomarker: N	Well tolerated
		Phase 3 NCT03053063 (STELLAR-4) [134] **TERMINATED**	Adult NASH with F4 883	**18 mg/day** OR **6 mg/day** OR Placebo	48, 240 weeks	Histology: X (Did not meet primary outcome during interim analysis at 48 weeks, hence terminated) Imaging: N Biomarker: N	
**Ceniciviroc (CVC)**	CCR2/5 antagonists	Phase 2b NCT02217475 (CENTAUR) [135]	Adult biopsy-proven NASH with F1-F3 289	**150 mg/day for 2 years** OR Placebo for 1 year followed by 150 mg/day for 2 years OR Placebo for 2 years	2 years	Histology: X (for the 1st year of CVC) Imaging: N Biomarker: X (ALT, AST, FIB-4, NAFLD fibrosis score, ELF)	Well tolerated
		Phase 3 NCT03028740 (141) **TERMINATED**	Adult biopsy-proven NASH with F2–F3 Part 1: 1200 Part 2: 800	**150 mg/day** OR Placebo	40 months	Histology: X (based on Part 1) Imaging: N Biomarker: X	

Legend: dosage. **Bold** = Drug(s) evaluated in results. Results. ✓ = Significant compared to placebo/non-bolded drugs; X = Not significant compared to placebo/non-bolded drugs; N = No results available.

### 8.2. ASK-1 Inhibitor

*Selonsertib (TERMINATED).* The activation of ASK-1 can result in worsening hepatic inflammation, fibrosis, and apoptosis [132,136]. As such, inhibition of ASK-1 is a potential intervention method for NASH. Selonsertib is a selective ASK-1 inhibitor and is also known as GS-4997. Previously, Gilead Science designed two phase 3 trials that evaluated the safety and efficacy of selonsertib in NASH patients with bridging fibrosis (STELLAR-3, NCT03053050) or NASH patients with compensated cirrhosis (STELLAR-4, NCT03053063) [134]. In both trials, the patients were randomly assigned to 6 mg/day selonsertib, 18 mg/day selonsertib, or placebo intervention for 48 weeks. Unfortunately, neither the 6 mg nor the 18 mg dose met the primary outcome of a significant improvement in fibrosis by one stage or more without worsening of NASH or the secondary outcomes of any fibrosis improvement or NASH resolution without worsening of fibrosis by the end of 48 weeks. In fact, the STELLAR-3 trial reported that both doses of the drug performed worse than the placebo. Only 12% of the 6 mg/day dose and 9% of the 18 mg/day dose achieved at least 1-stage improvement of fibrosis, compared to a higher 13% of the placebo group (Table 8). Meanwhile, the results were similar for STELLAR-4, with 14.4% in the 18 mg group and 12.5% in the 6 mg group achieving one or more fibrosis improvements after 48 weeks, which were not significantly better than those in the placebo group (12.8%). The adverse effects were similar among the selonsertib interventions, placebo, and earlier studies. Both trials were hence terminated early at week 48 due to insignificant primary histological outcomes, as stated in the Gilead Sciences study protocol.

The failure of these two drug trials demands careful evaluation of events leading to their failure to prevent another disaster [137]. One reason for failure stemmed from the lack of a successful placebo-controlled phase 2b trial to model the phase 3 trials after. Phase 2 trials can be split into phase 2a and phase 2b [137]. Phase 2a assesses what dosage is safe for patient administration, while phase 2b assesses drug efficacy at the prescribed dosage. The trial was modeled after a phase 2 trial [127] comparing simtuzumab and selonsertib instead, in which simtuzumab was treated as a placebo due to lack of efficacy in a previously terminated trial (NCT01672866) [138]. In fact, there was no significant difference in at least a 1-stage reduction in fibrosis between simtuzumab and selonsertib. Furthermore, the phase 2 trial pooled the results of selonsertib isotherapy and selonsertib-simtuzumab combination together, assuming a lack of synergy between the two drugs. Ignoring potential pharmacokinetics between selonsertib and simtuzumab could have led to disappointing results in STELLAR trials.

Although both trials failed to achieve primary outcomes, these clinical trials represent crucial opportunities to obtain important insights into the underlying biology of NAFLD therapeutics. For instance, clinical trials would put Gilead Sciences in a favorable position to explore systematic studies into precision medicine and generate high-throughput omics data and insights from patient samples. Subsequently, the establishment of a consortium of NAFLD therapeutics and associated omics deposits would monumentally provide greater access to elevate research in the translational NAFLD community. This can lead to a better understanding of the mechanisms that have led to failed trials.

### 8.3. CCR2/5 Antagonists

*Cenicriviroc (CVC) (TERMINATED)*. As a novel antagonist of CCR2/5, it has been shown in preclinical trials that CVC has antifibrotic effects in the kidneys and livers and can improve fibrosis and NASH [139,140,141]. CCR2/5 regulates the recruitment of monocytes and macrophages into the liver upon hepatic liver injury, which results in hepatic fibrosis and inflammation [142]. CVC inhibits CCR2/5 and eventually reduces monocyte/macrophage infiltration and, thus, collagen deposition, which plays a part in fibrogenesis. In the CENTAUR phase 2b study (NCT02217475) [135], the efficacy and safety of CVC, as well as the durability of the antifibrotic response beyond the 1st year, were evaluated in biopsy-proven NASH patients across 10 countries. The histological outcome was not met with a lower improvement in fibrosis by one or more stages with no worsening of NASH in CVC (12.8% in the 1st year, 15.2% in the 2nd year) compared to placebo (14.0% in the 1st year, 16.7% in the 2nd year). There was no significant histological improvement in at least one stage of fibrosis, with no worsening of NASH between CVC and placebo in the first year. In the 2nd year, there were also no significant improvements in primary outcomes. Furthermore, there was a decrease in patients achieving the secondary outcome in both groups. While the trial reported sustained antifibrotic findings, particularly in the advanced fibrosis patient subset of the CVC group, fibrosis improvement of at least one stage with no worsening of NASH deteriorated from the 1st year (20.0%) to the 2nd year (15.2%). There was also insignificant biomarker improvement in ALT, AST, FIB-4, NAFLD fibrosis score, and ELF in the CVC group compared to the placebo. CVC is well tolerated with common adverse effects, such as gastrointestinal, infectious, and musculoskeletal disorders (Table 8).

The trial combined results for patients treated with CVC for 2 years and placebo for the 1st year with CVC in the 2nd year. This could have both statistical and pharmacokinetic implications on the results comparing CVC and placebo in the 2nd year, which could be overcome by an increase in sample size.

Following this, AURORA was designed based on the CENTUAR trial. The AURORA phase 3 trial (NCT03028740) [143] studied the safety and efficacy of CVC in biopsy-proven NASH patients with F2–3. The study was split into two parts: Part 1 will study the outcome of at least one stage improvement in fibrosis with no worsening after 12 months; Part 2 will study the same group of individuals with newly added subjects to evaluate long-term CVC interventions in relation to histopathological progression to cirrhosis, liver-related outcomes, and all-cause mortality for 42 months. The adverse side effects will be assessed throughout the studies (parts 1 and 2). Unfortunately, the study was terminated early due to a lack of efficacy based on Part 1 results, according to ClinicalTrials.gov. The primary outcome of at least one stage of fibrosis improvement with no NASH worsening was not achieved, with a lower percentage of patients meeting the outcome in the CVC group (22.3%) compared to the placebo (25.5%). At least one stage of fibrosis improvement alone yielded lower results in the CVC group (30.3%) than in the placebo group (33.3%). The remaining histological secondary outcomes were not achieved either. The adverse effects in the CVC group were similar to those in previous studies.

## 9. Other Drugs for NAFLD

### 9.1. THR Agonist

*Resmetirom (MGL-3196)*. A novel thyroid hormone receptor beta-selective agonist oral drug (THR-β agonist) developed by Madrigal Pharmaceuticals was designed to treat NASH patients [12]. Resmetirom was previously shown to be specific to the liver and highlighted specifically with 28-fold more selectivity for THR-β over THR-α (Figure 3). This is promising, as the adverse effects of excess thyroid hormones are mediated by THR-α, while the lipid level effects are mediated by THR-β [144]. By having high specificity for THR-β, treatments using Resmetirom can be beneficial in enhancing hepatic fat metabolism as well as attenuating lipotoxicity in NAFLD patients without having to deal with the adverse effects of hyperthyroidism from THR-α stimulation. In addition, it is believed that there is a connection between hypothyroidism and NAFLD [145].

In Madrigal Pharmaceuticals’ phase 2 study (NCT02912260) [146], the efficacy of resemetirom in reducing hepatic fat in biopsy-proven noncirrhotic NASH patients was investigated. Significant histological improvements in a 2-point or greater reduction in NAS with at least a 1-point reduction in ballooning or inflammation were reported in the resemitron group compared to the placebo group. Significant improvement in hepatic fat measured via MRI-PDFF was also observed in the resmetirom group (−37.3%) compared to placebo (−8.5%). There were also significant biomarker improvements in ALT, AST, GGT, ELF, CK-18, adiponectin, LDL, and triglycerides compared to the placebo group.

Resemitrom was generally well tolerated, with mild to moderate side effects. The most common would-be nausea and diarrhea, with diarrhea occurring mostly at therapy initiation. No significant effects on thyroid-stimulating hormone levels, cardiac function, or diabetic markers were noted. Furthermore, the lipid-lowering effect of resmetirom can lower cardiovascular risk in NASH patients.

Looking forward, MAESTRO-NASH (NCT03900429) [147] is a phase 3 trial recruiting 2000 biopsy-proven NASH patients with F2–3. It is designed to study the efficacy and safety of Resmetirom at higher doses, as post hoc analysis of 80 mg/day and 100 mg/day showed greater NASH resolution, hepatic fat reduction, and lipid reduction (Table 9). Likewise, MAESTRO-NAFLD (NCT04197479) [147] is also a phase 3 trial that studies the safety and efficacy of Resmetirom in patients with NAFLD. Both studies are currently still ongoing.

### 9.2. FGF Analog

*NGM282 (phase 2)*. FGFs play a vital role in tissue development, maintenance, and repair by regulating cell proliferation and differentiation [147]. FGF19 is a postprandial hormone secreted from the small intestine via FXR receptor stimulation and is responsible for downregulating bile acid synthesis and gluconeogenesis while stimulating glycogen and protein synthesis [154].

NGM282 is a synthetic FGF19 analog (Figure 3). In a phase 2 study (NCT02443116) [148], the efficacy and safety of NGM28 were evaluated in biopsy-proven NASH patients with F1–3. The results showed at least a 5% reduction in absolute liver fat content measured by MRI-PDFF in 79% of the 6 mg NGM282 group and 74% of the 3 mg NGM282 group, achieving significantly higher results compared to 7% in the placebo group (Table 9). Complete normalization of liver fat was even achieved in both NGM 282 groups, with none in the placebo group. Significant biomarker improvements in ALT, AST, and triglycerides were also noted in the 6 mg NGM282 group compared to the placebo. The 3 mg NGM282 group reported significant improvements in pro-C3 (an indirect marker of collagen formation in fibrogenesis) and ELF compared to the placebo (Table 9). LDL concentrations, however, rose significantly in both NGM282 groups compared to placebo, which can increase the risk of cardiovascular mortality. NGM282 side effects are mostly mild, with injection site reactions, nausea, and gastrointestinal disorders being the most common [155].

Another open-label study [149] assessing histological efficacy was carried out with 43 patients with biopsy-confirmed NASH. Subcutaneous NGM282 (3 mg/day or 1 mg/day) was administered for 12 weeks. Histological results showed significant improvement in NAS by two points or more without fibrosis worsening in the 3 mg (68%) and 1 mg (50%) groups. Fibrosis improvement by one stage or more without worsening of NASH was also demonstrated in 42% of the 3 mg NGM282 group. Reductions in hepatic fat content and biomarkers such as pro-C3, ELF, AST, and ALT were also observed in both NGM282 groups.

These results occurred in the short span of 12 weeks, much faster than other trials normally running for >24 weeks. with the significant and rapid improvement in liver histology, fat, and biomarkers, as well as a well-tolerated profile, NGM282 proves itself to be a promising agent for NAFLD treatment. How long this antisteatotic effect can last with and without daily NGM282 administration is another question that must be answered through longer-term clinical trials. A future larger-scale phase 2 study [149] with 250 NASH patients is currently ongoing to determine the efficacy and safety of NGM282 in a larger sample size (Table 9).

*Pegbelfermin (phase 2).* FGF21 is highly expressed in the liver. It is responsible for glucose metabolism, lipid metabolism, and improving insulin sensitivity [156]. In recent years, studies have demonstrated potential FGF21 effects, such as anti-inflammation, β-oxidation, and apoptosis prevention [157]. While endogenous FGF21 has a short half-life of 1–2 h, synthetic FGF21 analogs have been created to prolong such effects. Pegbelfermin (BMS-986036) is a synthetic FGF21 analog with a long enough half-life to allow for weekly dosing (Figure 3). In a phase IIa study (NCT02413372) to investigate the safety and efficacy of pegbelfermin in NASH, 75 patients with biopsy-confirmed NASH were administered 20 mg/week, 10 mg/week pegbelfermin or placebo for 16 weeks [150]. There was a significant decrease in absolute hepatic fat as measured by MRI-PDFF in the 20 mg (−5.2%) and 10 mg (−6.8%) groups compared to the placebo (−1.3%), with half of the peglbelfermin groups achieving at least 30% relative hepatic fat reduction. Biomarker improvements were observed in ALT, AST, adiponectin, LDL, triglycerides, and PRO-C3 (Table 9). Notably, anti-FGF21 and anti-pegbelfermin antibodies were first detected on the 15th and 57th days, respectively, in both pegbelfermin groups. No immune-related adverse events or changes in pharmacokinetics or pharmacodynamics were noted, with antibody titers dropping 6 months posttreatment.

The side effects of pegbelfermin were mild, with most being gastrointestinal complications, such as diarrhea (16%). Currently, two other larger phase 2 trials, FALCON and FALCON 2 [151] are underway to test the efficacy of pegbelfermin in more advanced NASH patients with cirrhosis. No phase 3 trials have been confirmed yet.

### 9.3. Nutraceuticals

Stepping away from conventional drugs, nutraceuticals as a treatment for NAFLD may be an area of untapped potential. Defined as a food or dietary supplement providing health benefits to prevent or treat diseases, nutraceuticals are believed to have a blanket effect on the body’s metabolic processes, unlike the receptor-specific mechanism of action of conventional drugs [158]. Most nutraceuticals are more readily available and less costly than drugs, and being naturally occurring reduces the chances of adverse events [159]. While the main NAFLD management strategy involves lifestyle intervention through dietary changes, nutraceuticals are definitely an important area to look into for more concrete and evidence-based guidelines for managing NAFLD. Currently, a plethora of nutraceuticals has been explored, ranging from dietary extracts to herbal and traditional Chinese medicine origins. Although it is not the scope of this review to provide an exhaustive compilation of all the nutraceuticals (reviewed thoroughly in the other literature [160,161,162]), we highlight several key approaches and an overarching idea of the nutraceutical landscape in NAFLD intervention.

*Vitamin E.* Vitamin E is an antioxidant that protects cells against oxidative stress [163]. It can limit hepatocellular injury in NAFLD, which arises from ceramides acting on mitochondria to generate radicals such as reactive oxygen species and hydrogen peroxide. Vitamin E to date is the most researched nutraceutical in NAFLD management, yet the number of trials is still very limited compared to conventional drugs.

The PIVENS phase 3 trial (NCT00063622) [44] studied the effects of pioglitazone and vitamin E on hepatic histology in biopsy-proven NASH patients without T2DM. The primary outcome was met, with vitamin E demonstrating significant histological improvement in overall NAS compared to placebo. Fibrosis scores and NASH resolution were not significantly improved compared to the placebo. Significant biomarker improvements occurred in ALT and AST within the first 24 weeks compared to placebo but rapidly increased back to placebo levels after discontinuation. The certainty of relapse is high after therapy is ceased; hence, it is likely that vitamin E must be taken indefinitely. Adverse effects are minimal according to the trial but should be further investigated to determine vitamin E’s viability as a long-term therapy.

Another phase 2 trial [152] investigated the efficacy of vitamin E, UDCA, and PTX isotherapies in Egyptian patients with biopsy-proven NASH. There was significant biomarker improvement in IL6, CCL2/MCP-1 AST, and ALT in vitamin E compared to the other drugs. Patients also reported significant symptomatic relief of abdominal pain and malaise from baseline in the vitamin E group compared to the other drugs.

Combinations with vitamin E are more common than with any other potential NAFLD drug. A pilot trial [153] investigated the effects of vitamin E, PTX, and vitamin E-PTX combinations in biopsy-proven NASH patients. The primary outcome was not achieved, with no significant biomarker improvement of ALT in PTX-vitamin E compared to vitamin E isotherapy. Both groups showed improved overall NAS but were not significantly different from each other. Interestingly, a significant reduction in fibrosis occurred in PTX-vitamin E compared to vitamin E. There were significant biomarker improvements in HOMA-IR and TNF-a compared to vitamin E. Both groups showed imaging improvements in liver stiffness measured by FibroScan. No patients complained of side effects in vitamin E isotherapy, while 8.3% of patients complained of minimal gastrointestinal adverse effects in PTX-vitamin E.

There is promise in combination therapy, with PTX-vitamin E displaying improved fibrosis-reducing capabilities while maintaining vitamin E’s ability to improve overall NAS and biomarkers. However, compared to the initial biopsy number, only 19.5% of patients in the PTX-vitamin E group and 15.2% in the vitamin E group underwent repeat biopsies. Comparison of overall NAS and fibrosis score improvement may hence not yield significant results.

While synergistic effects are an important branch of drug therapy, current research should first focus on investigating the efficacy and safety of vitamin E as an isotherapy against a placebo to gain FDA approval.

*Fish oil (Lovasa).* Lovasa is used in the treatment of hypertriglyceridemia, with the primary mechanism appearing to be to reduce the production of triglycerides by hepatocytes. It is also found to decrease the hepatic production of VLDL and has antioxidant properties [154]. Due to this function of Lovasa, it is used to treat NAFLD and is the only fish oil supplement.

That has been approved by the FDA.

*Vitamin D.* Several clinical studies have also been carried out to determine the effectiveness of vitamin D in the treatment of NAFLD. Studies have shown that low serum vitamin D levels may lead to an increase in the severity and incidence of NAFLD and NASH. In this case, vitamin D supplementation was found to improve lipid profiles and inflammatory mediators [164]. However, there are also studies [165] that contradict this finding in finding that the high dose of vitamin D supplementation on NAFLD did not improve fatty liver content or histological parameters in NASH.

*Other nutraceuticals.* In recent years, although many clinical studies have been implemented to ascertain the effects of specific nutraceuticals on NAFLD patients, experimental designs (e.g., dosage and standardization of compounds) are heterogeneous and lack concrete conclusions. For instance, Silymarin, a mixture of extracted compounds from milk thistle *Silybum marianum*, is an extensively studied nutraceutical in NAFLD. In a recent systematic meta-analysis by Kalopitas et al., it was found that across seven RCTs included in the analysis, AST and ALT values consistently improved significantly in the patients. There were no substantial changes in body weight and no improvements in liver histology. Furthermore, the study pointed out several limitations in the designs of these RCTs, such as the high risk of bias in some studies and the lack of profiling of the ethnicities of the NAFLD patients [166]. Due to the lack of liver biopsy performed in most of the studies, the sample sizes for liver histology are small and insignificant. The dosage of Silymarin intervention used also varied drastically, varying from 140 mg/day to 2100 mg/day. Following these findings, Silymarin has been proposed as a recommendation by an expert panel in the Middle East for NAFLD patients with elevated liver enzyme levels for 3 months before reassessment with liver biochemistry tests [167].

Similarly, L-carnithine has been gaining attention recently. L-carnithine is an important amino acid derivative that is integral in transporting free fatty acids through carnithine shuttle systems [168]. with its intricate role in metabolism, L-carnithine has also been reported to be deficient in NAFLD patients. In preclinical animal models, L-carnithine supplementation in diet-induced NAFLD mice has been shown to ameliorate NAFLD and metabolic syndrome through the remodeling of metabolic and inflammatory dysregulation [169], as well as the regulation of gut microbiota. Clinically, a recent meta-analysis also revealed that L-carnithine reduced liver enzyme levels in chronic liver disease patients [170]. However, it was pointed out that most of these studies are combinatorial strategies using another drug, such as metformin, which has low sample sizes and is heterogeneous in its patients (obese and/or lean were combined, with differing outcomes) [171]. As such, the results have been conflicting in other aspects, such as improving insulin resistance, the bioavailability of L-carnithine in muscles, liver, and circulation (depending on delivery method) or even causing HCC [171] in high-fat diet-fed rodents.

Overall, the current nutraceutical field currently lacks standardization for safe and efficacious usage [172]. Furthermore, most studies mostly accounted for the improvement of liver enzyme levels and no other pathologies of NAFLD. Hence, future clinical studies on nutraceuticals require further investigation to assess the bioavailability of the supplement in patients [173]. In line with this, Manocha et al. proposed the use of nanoparticles for the specific and efficacious delivery of beneficial compounds in circulation [174]. Notably, the nutraceutical field is also shifting toward the use of synergistic compounds. For instance, a nutraceutical mixture of vitamin E, L-carnithine, and Silymarin was used as an interventional treatment. Interestingly, the mixture did not find improvement in liver enzyme levels but found improvement in metabolic syndromes, such as insulin resistance and dyslipidemia. However, there were also no histological or radiological assessments of the liver condition [175]. In another study, a synergistic combination of a curcumin complex, omega-3 PUFAs, BPF, artichoke leaf extract, black seed oil, and hepato-active compounds were used in the Livogen Plus mixture and successfully reduced liver fat content (via CAP), liver enzymes, and dyslipidemia in NAFLD patients [176]. Hence, with the increasing refinement of regulatory frameworks and clinical research in nutraceuticals, as well as the innovation of combinatorial synergistic nutraceutical mixtures, the nutraceutical field is expected to subsequently deliver interesting outcomes in the near future.

## 10. Targeting the Gut Microbiome

Gut microbiome dysbiosis is a significant risk factor for NAFLD [177]. Animal models have presented evidence for microbiota playing a causal role in the development of NAFLD, with studies showing that germ-free mice are protected against obesity and hepatic steatosis [178] and other studies showing that direct fecal microbiota transplant from mice with NAFLD to healthy mice results in the development of NAFLD [179]. While mechanistic and causative studies regarding the relationship between human gut microbiota and NAFLD are comparatively lacking, associations between the human gut microbiome and NAFLD have been proposed and are currently undergoing further research.

A proposed mechanism is that gut dysbiosis, and its associated changes in microbial metabolites and interactions result in increased permeability of the gut mucosal wall. This leads to increased absorption of free fatty acids and leakage of toxic bacterial-associated molecules, such as proinflammatory cytokines and lipopolysaccharides, which cause hepatic fat accumulation, inflammation, and injury [180]. The effects are the most prominent in the liver due to the liver’s close anatomical and functional relation to the gut. It is envisaged that with alteration of the gut microbiome, the healthy gut function can be established, and such biochemical processes can be reversed or attenuated, resulting in improvements in disease severity or progression of NAFLD. Several means of altering gut microbiota have been proposed and will be covered below, namely, probiotics, fecal microbiota transplant, and antibiotics.

*Probiotics, prebiotics, and synbiotics.* While studies show that probiotics result in improvements in liver function tests and lipid profiles of NAFLD patients, there is currently insufficient evidence that probiotics improve liver histology [181]. As such, further research into the effects of probiotics on liver histology is needed. Other considerations in evaluating the reliability of data regarding probiotic use include the sensitivity of the gut microbiome to variations in the diet and lifestyle of patients within the same study and significant variations in the dosage and preparation of probiotics between studies. Probiotics were defined by the World Health Organization (WHO) in 2002 as “live microorganisms that confer a health benefit when consumed in adequate amounts” [182]. While *Bifidobacterium* and *Lactobacillus* strains are the most prominently utilized probiotics in dietary supplements, other strains of probiotics, such as *Faecalibacterium prausnitzii and Akkermansia muciniphila,* are also actively being researched. [183] Probiotics exert their effects through the introduction of bacterial species associated with health benefits into the intestines, whereupon these bacteria function directly through the production of beneficial microbial products or by encouraging the growth of other beneficial species of bacteria. [184] with its colonization, these bacteria also concurrently reduce bacteria associated with disease states, aiding in the establishment of an ideal microbiome composition.

A 2019 meta-analysis [185] involving 782 patients from 15 RCTs also showed that probiotics and symbiotic supplementation could significantly improve markers of liver injury and biochemical contributors to NAFLD. These include reductions in liver steatosis, alanine aminotransferase, aspartate aminotransferase, triglyceride, total cholesterol, stiffness, and TNF-α. There was no significant effect on related risk factors for NAFLD, such as BMI, waist circumference, and fasting blood sugar [185].

Recently, a 2022 systemic review [186] involving 947 subjects from 13 randomized controlled trials (RCTs) studying the impact of probiotics, prebiotics, and synbiotics on markers of NAFLD and NASH concluded that although probiotics are not curative for NAFLD, probiotics could be used as adjuncts to reduce risk factors, thus aiding in slowing down pathological processes involved in disease progression and severity. Specifically, the trials analyzed showed that the consumption of probiotics, prebiotics, and synbiotics was associated with a reduction in risk factors for NAFLD, such as triglycerides, fasting insulin, fat percentage, and BMI. There was also a reduction in biochemical contributors to the pathophysiology of NAFLD, such as LPS, TNF-α, and IL-6. Measures of liver injury, such as AST, ALT, liver stiffness, fibrosis index, NAFLD, and NASH scores, also showed improvement. Some studies even captured an increase in bacteria from the phylum *Firmicutes*, which is known to contribute to the maintenance of gut mucosal integrity and hence reduces hepatic susceptibility to toxins and inflammation [186]. The apparent discrepancy between the two analyses underscores the importance of a more in-depth understanding of the impact of the gut microbiome on NAFLD and its risk factors.

*Fecal microbiota transplant (FMT)*. Fecal microbiota transplantation (FMT) is a nonspecific technique for altering gut microbiota and has been used successfully and safely in treating other diseases related to gut dysbiosis, such as *Clostridium difficile* infection. [187] In FMT, fecal matter is transferred from a healthy individual to a diseased individual’s gut, and although the exact mechanisms are still unclear, it is envisaged that subsequent colonization by the transplanted microbiome establishes a new gut microbiota community, restoring healthy gut function. [188] The procedure for FMT generally involves the collection of a fresh stool sample that is processed to remove solid particles and formulated into a ready-to-administer preparation for transplantation. This formulation is then administered into the recipient’s gut via various methods, such as colonoscopy, nasoenteric tube, gastroduodenoscopy, enema, or encapsulation for ingestion [187].

In a proof-of-principle study conducted in 2020 [189], it was found that FMT from a healthy donor contributed to histological improvements in NASH and increased the expression of protective genes in NAFLD progression. Furthermore, in a recent study, a 2022 randomized clinical trial [190] involving 75 patients with NAFLD conducted FMT via colonoscopy and enema. An evaluation of these patients 1 month later found that the FMT group of patients had reduced hepatic fat attenuation seen on FibroScan, but there were no significant differences in lipid levels or liver function tests between the FMT and non-FMT groups of patients. Despite these positive indications of therapeutic FMT in specifically reducing hepatic steatosis, the clinical trials had small sample sizes (n = 21, [189]), and improvements in hepatic injury were also not consistently found across all diagnostic markers, particularly in histology.

*Antibiotics.* Evidence regarding the use of antibiotics for NAFLD in humans is limited, as large-scale and clinical trial studies are lacking [181], and the risk of antibacterial drug resistance and side effects of antibiotics render their use less attractive. Of these, rifaximin is the more studied antibiotic in NAFLD clinical trials. However, mixed results have been observed in various studies. Previously, a phase 4 study in rifaximin and NAFLD (RiFL, NCT01355575) was terminated due to no effects in protecting the patients (n = 15) against hepatic fat accumulation. In contrast, a 2020 observational cohort study [191] found that rifaximin treatment resulted in a reduction in hepatic injury serum markers and steatosis, as well as a reduction in serum endotoxin and IL-10. Nonetheless, these studies indicate the need for further mechanistic and clinical investigations to yield more weighted conclusions.

## 11. Perspectives

### 11.1. Challenges in NAFLD Drug Development

While a large range of NAFLD pharmaceutical drugs is being tested, none are presently approved by the FDA. Drug development is subject to FDA approval before widespread distribution to patients for NAFLD treatment. Two regulatory pathways for drug development are (1) clinical outcome as a measure of clinical benefit based on patient quality of life or surrogate outcome that predicts clinical benefit on irreversible morbidity or mortality but is not itself a measure of clinical benefit or (2) accelerated approval pathway for serious life-threatening illness with surrogate outcome exceeding existing therapies [192]. In our opinion, these regulations cannot be met easily with the following challenges in the current design of clinical trials.

*Limited data.* Limited data are available for many potential NAFLD drugs. Data are collected through clinical trials that pass through successive phases differing in purpose, sample size, and length of study. Phase 1 tests up to 100 patients for several months to determine the safety and dosage of the drug. Phase 2 tests of up to several hundred patients for up to 2 years were performed to determine the efficacy and side effects of the drug. Phase 3 studies of up to 3000 patients for up to 4 years were performed to determine the longer-term efficacy and side effects of the drug. Finally, phase 4 studies of up to several thousand patients were conducted to obtain the widest range of data on the efficacy and side effects of drugs. To date, the majority of current NAFLD drugs are only in phase 2 trials, with a handful having completed a phase 3 trial. Only pioglitazone and vitamin E have completed phase 4 trials and have yet to receive approval from the FDA. We recognize practical challenges such as patent expiration and limited funding capabilities as barriers to conducting more trials by a single pharmaceutical company. One way to facilitate drug development is to expand these trials into clinical studies in collaboration with the basic science community. For instance, deep omics profiling of patient samples has been performed for various cancers and deposited in online public databases such as Gene Expression Omnibus (GEO), ArrayExpress, and The Cancer Genome Atlas. Such datasets have been used by basic scientists to reclassify bladder cancer patients based on the molecular phenotypes of responses to treatment [193]. In AML, an in silico prediction model has been implemented to identify patient subtypes of specific polygenic biomarkers that are responsive to combination therapy of mitoxantrone, etoposide, and cytarabine. [194] These studies were possible due to collaborative work with basic scientists to profile patients before and after treatment, as well as the establishment of online resources for data accessibility. In NAFLD therapeutic development, there is a severe lack of such experimental designs and patient profiling in the basic science scene, as well as established online resources for standardized data sharing.

*Challenging trial design and outcomes*. An indication for termination includes not meeting FDA-approved outcomes, which include all-cause mortality and histological improvement. These outcomes are approved as the most reliable measure of clinical benefit to a patient’s quality of life. All-cause mortality has long been established as one of the most important clinical outcomes in clinical drug development trials. As a subcomponent, liver-related mortality is a critical factor to study. Ideally, larger sample size studies with early-stage NASH and more than 15 years of longitudinal studies can be carried out. However, it is difficult for companies to fund wide-scale long-term trials that are particularly capital-intensive. Considering less resource-intensive strategies for data collection, such as the standardization of noninvasive outcomes over biopsy, is a possibility to make trials more economically viable. However, more time is required for noninvasive outcome development and widespread implementation.

Liver biopsy remains the gold standard surrogate outcome to determine the progression or regression of NAFLD. For advancement into phase 2b or three trials, at least 1 of the following two surrogates must be met: (1) NASH resolution without fibrosis worsening or (2) fibrosis improvement by at least one stage without NASH worsening [90]. However, not all studies included fibrosis improvement, overall NAS improvement, or NASH resolution in their primary or secondary outcomes. Some trials have even had potentially skewed results due to the excessive risk of biopsies [135]. As biopsies are one of the most expensive investigation methods, most of the current studies do not have sufficient funds to biopsy a larger sample size of patients. Furthermore, the invasive nature of biopsies makes the threshold for indication higher than other investigations. This poses a problem for many NAFLD patients who are asymptomatic or at the early stages of NAFLD, as a liver biopsy may do more harm than benefit. In the PTX-vitamin E trial, a small fraction of PTX-vitamin E (19.5%) and vitamin E (15.2%) patients underwent repeat biopsies after a year compared to the initial biopsy number, potentially underpowering results collection. Liver biopsies are also subject to random sampling variability, with only a small section of the liver analyzed without revealing the general status of the whole liver. with this limitation on the number of studies able to carry out a liver biopsy, conditional approval becomes more difficult to attain for most drugs. Looking forward, increased reliance on noninvasive and responsive markers as measures of all-cause mortality and liver biopsies will pave the way for accurate, larger-scale, yet shorter-term clinical trials.

*Debilitating adverse events.* Another indication for termination is adverse events. Adverse events are actively evaluated and monitored in clinical trials and can even warrant early termination in the best interest of the patients if deemed too severe. Adverse events will be assessed as mild, moderate, or serious, and causal relationships will be determined. Pioglitazone and OCA, both drugs in phase 3 and beyond in clinical trials, have yet to receive approval from the FDA due to adverse effects reported. While mild to moderate in nature, a higher than the tolerated proportion of patients suffering from adverse effects, making it difficult to distribute widely as a recommended drug. Hence, employing different strategies (Section 11.2) to manage any risks involved will be a key factor in drug approval.

### 11.2. What Can We Learn from NAFLD Clinical Trials?

With the overwhelming number of clinical trials carried out each year, it is important to take a step back and reflect on the lessons learned behind every success and failure.

*Drug repurposing*. Repurposing drugs is a quicker solution to identifying the right NAFLD drug therapy. Novel drugs are required to undergo all 4 stages of FDA drug approval, which is very time consuming and resource-heavy. On the other hand, existing drugs that have been profiled for efficacy and safety can be approved much faster. As they are already prescribed to many patients, the tolerability of the drug can be assured. Furthermore, these drugs have already proven therapeutic benefits for a particular condition. If the targeted condition is also a common risk factor or complication of NAFLD, said condition and NAFLD can both be managed with a single drug. Additionally, drug trials are easier, given previous large-scale clinical trials. Retrospective studies can be used, saving on cost and not compromising patient safety.

Considering common comorbidities and risk factors for NAFLD, potential candidates include OHGA, statins, and ezetimibe. For T2DM patients, pioglitazone is the most promising thus far, having undergone phase 3 and 4 trials. Along with lifestyle modifications and supplements such as vitamin E, both T2DM and NAFLD can be concurrently managed. For patients with dyslipidemia, statins and ezetimibe have yet to undergo phase 3 trials. However, ezetimibe has shown potential in direct NAFLD management with histological improvement, while statins can be used as an adjunct therapy to reduce cardiovascular risk in NAFLD patients [195].

However, there are challenges to drug repurposing, as previously mentioned, namely, company patent expiration during the clinical trial phase and the economic burden it bears on that single company if the drug is not readily available for research by third parties.

*Drug combinations*. Drug combinations are a promising area for investigation. The multifactorial nature of NAFLD, along with the different stages of NAFLD progression, means that a single miracle drug does not exist. Different drugs with different pharmacokinetics are required to target risk factors and mechanisms. Clinical trials have shown that most drugs excel in achieving different clinical outcomes, be it overall NAS improvement, fibrosis improvement, and more. Vitamin E-pioglitazone demonstrated a better histological improvement within a shorter period than pioglitazone isotherapy. Here, pioglitazone has been shown to promote histological improvement in NAFLD patients, while synergistic effects from vitamin E could have amplified it. Furthermore, the side effects of an individual drug can be alleviated with a combination with another NAFLD drug therapy. Vitamin E-pioglitazone can be paired with lipid-lowering drugs such as statins to combat its side effects of increasing cholesterol levels and increased cardiovascular mortality. Understandably, drug combinations can introduce a slew of adverse reactions between drugs or patient comorbidities. However, if trailed successfully in the right patient demographics, successful results in a shorter time with reduced side effects can hasten drug approval. From the results gathered, this paper finds vitamin E-pioglitazone, PTX-fenofibrate, and PTX-vitamin E to have achieved the most positive results in NAFLD improvement.

*Adverse event alleviation*. All drugs give rise to adverse events, only differing in severity and proportion of patients affected. Currently, drug-related adverse events have better morbidity and mortality rates than current management options, such as invasive bariatric surgery. Rebound NAFLD is also risked in patients who are nonadherent to lifestyle changes, especially strict diets for weight loss. However, what matters most in drug trial approval is whether the patient can tolerate the side effect. Side effect tolerance is very low given the low-risk profile of most NAFLD patients; hence, drugs such as pioglitazone, which is an approved T2DM medication, have yet to be approved due to side effects. Hence, balancing benefit and risk through intervention is crucial, and this can be conducted via the implementation of the Risk Management and Mitigation Strategy (REMS) [196]. REMS ensures that certain actions are taken to prevent and monitor adverse events to reduce their severity and frequency.

By taking pioglitazone as an example, each adverse event can be addressed by defining novel guidelines to mitigate these effects. Peripheral edema due to fluid retention is especially worrisome in NAFLD patients with a high risk of cardiovascular complications. However, most patients without end-stage kidney disease respond well to low dosages of diuretics, such as thiazides and spironolactone, which have been proven to reduce the risk of fluid overload with daily use. Diuretics are also indicated in congestive heart disease management, which could lower the risk of cardiovascular side effects by pioglitazones in NAFLD patients [197].

Weight gain is an understandable side effect due to the expansion of peripheral adipocyte storage and fluid retention. However, as fat gain occurs mainly in subcutaneous tissues instead of visceral organs, this can instead stimulate a further increase in insulin sensitivity [198]. Furthermore, appropriate dosing with concomitant diet and exercise regimes can regulate weight gain issues.

Osteoporosis has been proposed to occur due to the differentiation of osteoblasts into adipocytes, reducing bone density and increasing the risk of fractures in distal extremities (forearm, wrist, tibia, foot) [199]. This is reported to affect postmenopausal women and the elderly more severely, and the risk increases with dosage and duration of treatment. Annual DEXA scan monitoring and encouraging a calcium-rich diet can help minimize the risks of osteoporosis. However, the epidemiologic shift in NAFLD to younger patients <45 years old means that the target patient group will be less at risk of osteoporosis. Furthermore, combined therapy with other NAFLD medications may halve the dosage of pioglitazone. Alternatively, combined therapy with bisphosphonates can minimize bone loss, with a study with mice showing alendronate completely negating bone loss that occurs in pioglitazone. This requires further investigation into how osteoporosis can be minimized as a side effect in T2DM patients.

If patients still do not respond well or have contraindications to said medications, dose titration strategies can also be employed. This includes delaying up titrations, temporarily down titrations and temporarily stopping intake with a slower up-titration. Synergistic drug combinations such as pioglitazone-vitamin E can also help to lower the administered dosage of both drugs. Such strategies can be considered for future clinical trials to investigate their efficacy and safety alongside the target drug. If successful, we will be one step closer to our first FDA-approved NAFLD drug.

*Noninvasive outcome standardization*. Outcomes serve as the determinant of success in clinical trials. It also indicates which measure is believed to be the best predictor of NAFLD diagnosis, severity, regression, morbidity, and mortality. Hence, it is important that the right outcomes are investigated using the most effective and relevant measures.

Most primary outcomes are based on histological improvements in either overall NAS improvements or fibrosis improvements. Three-quarters of the current outcomes require biopsies despite running a worse risk-benefit ratio against early-stage disease patients according to FDA guidelines [192]. NAS improvements focus on reduction in steatosis, inflammation, and ballooning, which are key factors in early-stage NAFLD progression. Fibrosis develops more frequently in late-stage NAFLD, leading to cirrhosis and liver-related complications. While NAS improvements are important, studies have shown that fibrosis is the best indicator of liver-related mortality. A liver with cirrhosis in an early-stage NAFLD patient is more likely to develop HCC and liver-related complications than a late-stage NAFLD without cirrhosis. Steatosis, inflammation, and ballooning can be reduced while fibrosis advances in late-stage NAFLD, making NAS improvements alone an inaccurate marker for mortality. A renewed and stronger focus on antifibrotics may be necessary. However, steatohepatitis remains the main driver of NAFLD. Pure antifibrotic treatment is insufficient for completely managing NAFLD, even for advanced NASH with cirrhosis. A potential recommendation is switching to drugs with strong anti-steatohepatitis effects after antifibrotic use to reduce fibrosis in NAFLD patients.

Currently, the above outcomes can be monitored by noninvasive measures but are ultimately confirmed only by histology. The pitfalls of histology have been previously discussed, and there is an obvious need for increased reliance on noninvasive methods of diagnosis and prognosis. Trials can focus on investigating the accuracy of US and CT techniques in predicting liver histology rather than using expensive and less commercially available MRI techniques.

Hepatic triglycerides, ALT/AST, and CK18 are also noninvasive hepatic measures directly related to the liver that are objective, quantifiable, and sensitive to change. However, additional validation is required for these markers to be used in late-phase trials. Currently, a select few early-phase trials and close to no late-phase trials utilize these biomarkers as primary outcomes. If more results are gathered that strongly associate these liver-related biomarkers with histological improvements in NAFLD, larger-scale trials can potentially be run at lower costs, and low-risk patients can be tested repeatedly for more accurate data collection, advancing the likelihood of drug approval. An example would be CK18, which has been associated with histological improvements in both adults and children with NASH in two phase 2 studies [200].

Systemic nonhepatic biomarkers such as HOMA-IR, HbA1c, LDL-C, and HDL-C are equally important given the multifactorial nature of NAFLD. Discovering an algorithm for reading biomarkers instead of basing histological improvements on a single biomarker could be a significant step in the right direction. An example would be staging histology using ALT, ALT, anti-inflammatory markers such as IL-23 to predict inflammation, hepatic steatosis index (HSI) to predict steatosis, and serum fucosylated haptoglobin, a novel diagnostic biomarker, to predict ballooning [200,201,202]. Patient quality of life is another noninvasive measure to use for monitoring disease severity. This can include emergency department admissions, hospitalization rates, unscheduled clinic visits, symptom flares, and even reduced requirements for biopsies. This is especially useful for a chronic disease with high retained functionality, whereby continuous noninvasive low-risk monitoring for a prolonged period can prove the drug’s true efficacy.

Looking forward, standardizing a set guide of noninvasive outcomes for future clinical trials to follow has the possibility to make large-scale trials not only more affordable and lower-risk but will also make trials more clinically relevant by screening for asymptomatic low-risk patients for treatment or to undergo prophylaxis against NAFLD. It is important that different stages can be recruited with said measures and are followed through closely. Having a standard set of values to compare across trials will allow drug development to progress with more relevant data available.

*Improvements in recruitment phase*. Current clinical trial models have the capacity to be improved on. This includes how the patients are recruited for trials. By starting at earlier phase trials, definitions and inclusion/exclusion criteria can be more stringent. Cirrhosis (F4) should be considered in a different category from bridging fibrosis (F1-F3). Patients with cirrhosis have a 6x higher risk of major cardiovascular side effects than bridging fibrosis; hence, more stringent stratification can help accurately predict drug efficacy to increase the likelihood of designing a successful clinical trial. Definition inconsistencies can also affect how data are interpreted. In the inclusion criteria, NASH included patients with fibrosis from F1-F4. In the primary/secondary outcome, NASH resolution only involves inflammation and ballooning markers reduced to 0 based on NASH CRN. Hence, even if the patient has a heavily fibrosed liver, NASH can still be considered resolved and overestimates the results.

Sample population enrichment can also increase the likelihood of more favorable results. Histological baselines such as ballooning, and steatosis level are normally used but are limited by higher variability among other patients as well as the invasive nature of biopsies. Recruiting patients with HPV >10 mmHg, MELD >10, high FIB4 score, T2DM, and fibrosis-related single nuclear polymorphisms (SNPs) can help induce higher patient responsiveness to drugs, ensuring that results are not underpowered. Using noninvasive parameters that are normally raised in NAFLD can ensure a higher-than-normal baseline for all patients recruited despite higher sample populations.

While phase 3 and 4 trials are modeled after earlier phases, seemingly minor changes can still be made to drastically affect the outcome. In this case, patient recruitment and inclusion criteria can be fine-tuned. Most NAFLD clinical trials consist of biopsy-proven NASH patients with F1–4. While it is useful to investigate management in advanced NASH with fibrosis, early identification and treatment are gaining more traction given the better mortality and morbidity outcomes. Additionally, the recruitment requirement of histological confirmation of NASH, although pathologically accurate, may need to change in today’s increasing reliance on noninvasive means for NAFLD diagnosis. This will allow clinical trials to become increasingly relevant to real-life clinical scenarios.

*Adoption of master protocol trial designs.* It is becoming increasingly clear that NAFLD is a highly heterogeneous disease with multiple pathogenic processes. Hence, future studies regarding NAFLD should shift away from traditional approaches of seeking a blanket therapy for the disease and instead seek to tackle the various sub-phenotypes of NAFLD with targeted therapies. As such, future trials would benefit from stratifying patients more extensively according to significant covariates that might suggest a different driving mechanism behind their development of NAFLD. NAFLD is the result of an interplay between genetic, metabolic health, and environmental factors, and further mechanistic research will be required to both discover and feasibly screen for specific biotargets for drug therapy for NAFLD. As a first step, studies could consider profiling and stratifying patients in detail according to covariates that currently have evidence affecting the development of NAFLD. Such covariates include age [203], sex [204], ethnicity, obesity as opposed to lean NAFLD [205], metabolic comorbidities [206], genetic and epigenetic factors [207], etc. This notion is increasingly being recognized, as further emphasized by the recent debates to rename NAFLD to metabolic dysfunction-associated fatty liver disease (MAFLD) [206]. The proposal of “MAFLD” terminology represents the shift toward the emphasis of considering associated metabolic dysregulation when evaluating NAFLD while recognizing the major subtypes of metabolic disorders in NAFLD: (i) obesity, (ii) T2DM, and (iii) lean metabolic syndrome. “MAFLD” is not used in this review article to maintain uniformity and correspondence to the main terminology (NAFLD) used in the literature review. While NAFLD patients are largely recruited and stratified based on histological grading of the extent of liver steatosis, inflammation, and/or fibrosis, MAFLD patients would be recruited based on underlying metabolic disorder subtypes that reflect differences in pathomechanisms and drivers of disease [208]. Hence, a greater focus on covariates of NAFLD enables greater precision in targeting patients with similar subtypes.

As such, NAFLD trials should aim to gather biochemistry data on all their participants according to a standardized list of specific and comprehensive criteria. This would enable investigators to regroup data according to research interest, allowing the data to be analyzed beyond the scope of the paper and allowing for better comparisons with data from other studies. Ampuero et al. [209] identified metabolic comorbidities of patients as key factors that significantly impacted the efficacy of drugs and proposed a standard list of relevant comorbidities. Practically, the use of the MAFLD framework may be a starting point for the collection of robust patient metadata. In the NHANES III cohort, it was found that 8.6% of patients were excluded due to missing biochemistry tests such as triglyceride, HDL, and high sensitivity-C-reactive protein (hs-CRP) levels, while 35.2% were excluded due to lack of insulin/glucose fasting tests [210]. The lack of these data prevented the comprehensive diagnosis of metabolic syndrome-associated MAFLD in lean patients. Hence, this suggests that even in the new MAFLD framework, many metabolism-related biochemistry tests were not conventionally performed for NAFLD, indicating a severe lack of standardization in these patient tests for precise interrogation.

**Figure 4:**
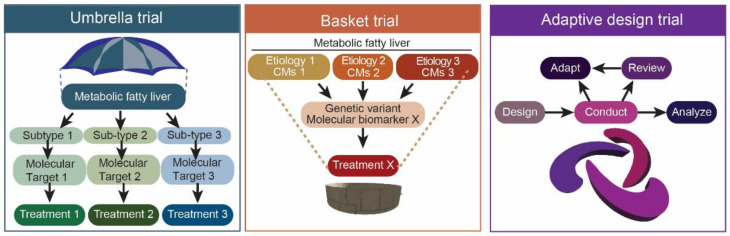
Schematic of Master Protocols application in NAFLD clinical trials. CM—comorbidities. Adapted from [206].

Furthermore, the adoption of novel trial designs, such as master protocols, may present a good option for better consideration of the subphenotypes of NAFLD, among other benefits. Master protocols are defined as overarching protocols designed to answer multiple research questions. The three most well-characterized designs under this definition of master protocols are umbrella, basket, and adaptive platform trials [211] (Figure 4). Master protocol methodologies have been well applied in various other fields, such as oncology, influenza, and Alzheimer’s disease [211]. Although master protocols have yet to be extensively applied in NAFLD, there have been several calls for a paradigm shift in this direction [208,212].

Umbrella trials involve screening and subsequent stratification of patients into different arms based on their subphenotype or relevant biomarkers of NAFLD, with each arm testing a different treatment that is targeted to it (Figure 4). NAFLD patients were stratified according to covariates which represent their various sub-types. Effectively, an umbrella trial takes the form of standardized and systematic conduction of many small trials within a large trial [209]. In addition to allowing more efficient stratification according to covariates, it provides several benefits, such as allowing for direct comparisons of multiple treatment options for disease and having a single control group that could be shared and used as a standard comparison for multiple different investigations. For instance, a recent proposal by Valenzuela-Vallejo et al. (2022) [213] proposed the reconsideration of NAFLD guidelines but rather an umbrella subtyping of FLD toward the repurposing of FDA-approved drugs against other metabolic diseases. Although an umbrella trial was not performed, the authors applied the umbrella principle to reconsider NAFLD guidelines based on other metabolic disorders, such as T2DM and obesity, as well as CVD outcomes. Consequently, this enabled the systematic justification to introduce novel drugs such as tirzepatide to be considered potential candidates only in specifically obese-related NAFLD patients. Subsequently, the standardization and repetition of the umbrella trial protocol would improve trial efficiency and speed of progress. However, it does have some downsides, such as the need for a centralized screening infrastructure to be established, as individually conducted screening (e.g., genotyping) may be less reproducible.

Basket trials identify and target a common biomarker that may be present in the pathogenesis of several related diseases. Hence, patients with different but related diseases, such as type 2 diabetes, NAFLD, and some cardiovascular diseases, identified to have a specific biomarker could be grouped together in the trial of a specific drug. Such biomarkers may be shared pathophysiological pathways, such as proatherogenic lipid alteration [214], insulin resistance with particular attention to the DAG-PKCε hypothesis [215], low-grade inflammation [216], and microbiome alteration [180], or novel and emerging targets based on established mechanistic knowledge, such as the various PPAR strains or MGAT enzyme expression [215]. Basket trials would be more efficient than conducting individual trials and can help save costs while speeding up the approval of new therapies. Pooling of patient numbers across diseases might also enable the collection of more safety data and promote a better understanding of the mechanisms behind the sensitivity and resistance to drug targets. Furthermore, with an innovative approach, a modified basket trial concept was applied to NAFLD preclinical models to discover a novel dipeptidyl peptidase-4 inhibitor-specific biomarker [217]. In this case, heterogeneous NAFLD presentations were induced with different diet-induced NAFLD rodent models, and a pretreatment biopsy was used to identify NAFLD rodents with high IGFBP-1 expression. Subsequently, treatment with a DPP-4 inhibitor indicated a higher response in these rodents. Such innovative approaches can subsequently provide proofs-of-concept for clinical translation. In this case, heterogeneity within NAFLD itself is integral in the design, where similar molecular therapeutic targets are identified from a basket of highly different NAFLD patients (defined through etiology, comorbidities, etc.). Subsequently, the patients are grouped according to these metabolic sub-types, and their targetable biomarker is identified and treated with its associated treatments (Figure 4). However, basket trials present difficulties in dosing and safety standards of the drugs, especially in their variability between diseases. Moreover, heterogeneity is inevitably introduced due to the nature of the trial design. The complexity of basket trials may also result in lengthy and complex protocols, which adversely affects the ease of running the trial.

In adaptive platform trials, patients are screened and stratified into different arms based on their subphenotype or relevant biomarker of NAFLD but have the added flexibility of “adapting” to findings earlier on in the trial by adding or stopping treatment arms [211]. Examples of such adaptive features include the addition of new therapies or populations at certain junctions during the trial or the halting of certain arms of the trial due to futility or side effects and reassigning patients to other arms (Figure 4). Adaptive platform trials have the benefit of streamlining the process of identifying patient subgroups that would benefit the most. For example, should early data show that patients who have NAFLD concurrently with type 2 diabetes experience milder side effects or experience enhanced efficacy to a particular drug (which was apparently the case with pioglitazone), more diabetic patients can be recruited to the trial, increasing the chances of the trial succeeding [209]. Patient subgroup arms that seem to have little or even adverse reactions to the treatment can be stopped at an earlier stage in the trial as well, minimizing any futility or harm caused to patients. However, this adaptive trial design adds significant complexity to not just the protocol but also to data analysis. It will require extensive infrastructure setup and long-term investment by relevant stakeholders.

## Figures and Tables

**Table 1:** NAFLD Activity Score (NAS) and Fibrosis Score. Adapted from [34].

NAFLD Activity Score			
Score	Steatosis	Lobular Inflammation	Ballooning Degeneration
0	<5%	None	None
1	5–33%	<2 foci/20× field	Few
2	>33–66%	2–4 foci/20× field	Many
3	>60%	>4 foci/20× field	
**Fibrosis Score**			
Stage	Histological Findings		
1a	mild pericellular fibrosis (only seen on connective tissue stain)		
1b	moderate pericellular fibrosis (readily seen on H&E)		
1c	portal/periportal fibrosis without pericellular fibrosis		
2	pericelluar and portal/periportal fibrosis		
3	bridging fibrosis		
4	cirrhosis		

NASH resolution is defined by three criteria: ballooning = 0, inflammation = 0 or 1, and no worsening of fibrosis.

**Table 2:** Steatosis, Activity, and Fibrosis (SAF) score. Adapted from [35].

SAF (Steatosis, Activity, Fibrosis) Score			
Score	Steatosis		
0	<5%		
1	5–33%		
2	>33–66%		
3	>60%		
Activity	Score	Lobular Inflammation (LI)	Ballooning Degeneration (BD)
A0–4	0	none	none
(LI + BD)	1	<2 foci/20× field	hepatocytes with rounded shape and pale cytoplasm usually reticulated. size is quite similar to normal hepatocytes.
	2	>2 foci/20× field	hepatocytes with rounded shape and pale cytoplasm usually reticulated. some cells are twice the size of normal hepatocytes.
Fibrosis	Histological Findings		
1a	mild pericellular fibrosis (only seen on connective tissue stain)		
1b	moderate pericellular fibrosis (readily seen on H&E)		
1c	portal/periportal fibrosis without pericellular fibrosis		
2	pericelluar and portal/periportal fibrosis		
3	bridging fibrosis		
4	cirrhosis		

**Table 3:** Summary of the current recommended interventions for NAFLD (NAFL and NASH) according to the NICE, EASL, and AASLD guidelines.

Intervention	NAFL	NASH
Lifestyle Modification	Yes	Yes
Pharmacotherapy	No	Yes, although pediatric differs among guidelines
Bariatric surgery	Yes, if obese	Yes, if obese
Liver Transplantation	No	Yes, only with end-stage liver disease such as liver failure

**Table 4:** Use cases of pharmacological treatment according to various guidelines globally (NICE, EASL, and AASLD).

Guidelines	Pharmacotherapy	Considerations	Therapy Duration	Care Settings
National Institute for Health Care and Excellence	Pioglitazone or Vitamin E	Adults, advanced fibrosis, with or without diabetes	24 months	Secondary to Tertiary
National Institute for Health Care and Excellence	Vitamin E	Pediatric, advanced fibrosis, with or without diabetes	24 months	Tertiary
European Association for the Study of the Liver	Pioglitazone or Vitamin E or combination	Advanced fibrosis (F2 or higher), NASH	6 months	Unspecified
American Association for the Study of Liver Diseases	Pioglitazone	Biopsy-proven NASH, with or without diabetes	Unspecified	Unspecified

Currently, the recommended pharmacological treatments across the guidelines for NAFLD are pioglitazone and/or vitamin E.

**Table 5:** Clinical trials on oral hypoglycemic agents were evaluated.

Oral Hypoglycemic Agents							
Drug	Mechanism of Action	Trial (Type and Identifier)	Patient (Type and Number)	Dosage	Duration	Results (Histology, Imaging, Biomarker) ✓ X	Side Effects
**Pioglitazones**	PPARγ agonist	Phase 4 NCT00994682 (UTHSCSA) [42]	Adult NASH 176	**45 mg/day with 500 kcal deficit** OR Placebo with 500 kcal deficit	18 months, 36 months	Histology: ✓ Imaging: X Biomarker: ✓ (adipose insulin sensitivity)	Long-term osteoporosis, weight gain, fluid retention
		Phase 4 NCT01002547 [43]	Adult NASH with T2DM 105	**45 mg/day with 800 mg/day Vitamin E and 500 kcal deficit** OR 800IU/day Vitamin E with 500 kcal deficit OR Placebo with 500 kcal deficit	18 months	Histology: ✓ Imaging: X Biomarker: X	Mild hypoglycemia, dyspnea
		Phase 3 NCT00063622 (PIVENS) [44]	Adult NASH 247	**30 mg/day** OR 800IU/day Vitamin E OR Placebo	24 months	Histology: ✓ Imaging: N Biomarker: N	
		Phase 4 NCT02365233 [45]		Pioglitazone or Lantus Insulin, DPP4 inhibitor Saxagliptin		Pending	
		Phase 4 NCT03910361 [46]		Pioglitazone or Evogliptin		Pending	
		Phase 4 NCT03796975 [46]		Pioglitazone + Metformin or Metformin		Pending	
		Phase 4 NCT03646292 [46]		Pioglitazone or Empagliflozin or both		Pending	
		Phase 3 NCT02265276 [46]		Pioglitazone or Saroglitazar		Pending	
**MSDC-0602K**	MPC inhibition	Phase 2 NCT02784444 (EMMINENCE) [47]	Adult NASH with F1-F3 392	**250 mg/day** OR **125 mg/day** OR 62.5 mg/day OR Placebo	12 months	Histology: X Imaging: N Biomarker: ✓	Mild gastrointestinal disorders, peripheral edema, weight gain
		Phase 3 NCT03970031 [48]	Adult NAFLD with pre-T2DM or T2DM and macrovascular cardiovascular disease 1800	62.5 mg/day OR Placebo	26 weeks, 15 months	Pending (September 2024)	
**Lanifibranor**	Pan-PPAR agonist	Phase 2 NCT03008070 (NATIVE) [49]	Adult NASH 247	**1200 mg/day** OR 800 mg/day OR Placebo	24 weeks	Histology: ✓ Imaging: ✓ Biomarker: ✓ (HbA1c, fasting plasma glucose, HOMA-IR and adiponectin)	Mild gastrointestinal disorders, peripheral edema, weight gain
		Phase 3 NCT04849728 [50]	Adult noncirrhotic NASH with F2–F3 2000	1200 mg/day OR 800 mg/day OR Placebo	72 weeks, 7 years	Pending (September 2028)	
**Saroglitazar**	Dual PPARα/γ agonist	Phase 2 NCT03061721 [51]	US or biopsy-proven NAFLD 106	**4 mg/day** OR **2 mg/day** OR **1 mg/day** OR Placebo	16 weeks	Histology: X Imaging: ✓ (MRI-PDFF, only 4 mg/day) Biomarker: ✓ (ALT, AST, ELF, APRI, triglycerides, VLDL, HOMA-IR)	Well-tolerated
		Phase 2 [52]	Biopsy-proven NASH 16	**4 mg/day** OR **2 mg/day** OR Placebo	24 weeks	Histology: X Imaging: N Biomarker: ✓ (LDL-C)	
		Phase 3 NCT04193982 [53]				Pending	
		Phase 2 NCT05011305 [46]				Pending	
**Elafibranor**	Dual-PPAR α/β agonist	Phase 2 NCT01694849 [54]	Adult NASH 270	**120 mg/day** OR 80 mg/day OR Placebo	56 weeks	Histology: ✓ Imaging: N Biomarker: ✓ (ALT, AST, HbA1c, triglycerides, LDL cholesterol, HOMA-IR)	Well tolerated
		Phase 3 NCT02704403 (RESOLVE-IT) [55] **TERMINATED**	Adult NASH with F1–3 2157	**120 mg/day** OR Placebo	72 weeks, 54 months	Histology: X (at 72 weeks, resulting in termination) Imaging: N Biomarker: X	
**Dapagliflozin**	SGLT2 inhibitor	Phase 2 [56]	NAFLD with T2DM 38	**10 mg/day** OR Placebo	12 weeks	Histology: N Imaging: ✓ (non-contrast CT) Biomarker: ✓ (ALT, HbA1c)	Well tolerated
		Phase 2 [57]	NAFLD with T2DM 57	**5 mg/day** OR Placebo	24 weeks	Histology: N Imaging: N Biomarker: ✓ (AST, ALT, HOMA-IR)	
		Phase 4 NCT02637973	Adult NAFLD with T2DM	25 mg/day OR Placebo		Pending	
		Phase 4 NCT02964715	Adult NASH with T2DM	25 mg/day OR Placebo		Pending	
		Phase 4 NCT03646292	Adult NAFLD with T2DM	10 mg/day OR 15 mg/day pioglitazone OR Both		Pending	
		Phase 4 NCT04642261	Adult NAFLD	10 mg/day OR Placebo		Pending	
		Phase 3 NCT03723252 (DEAN) [58]	Adult NASH with T2DM	10 mg/day OR Placebo		Pending	
**Empagliflozin**	SGLT2 inhibitor	Phase 3 IRCT20190122042450N1 [59]	Adult NAFLD with T2DM 91	**10 mg/day** OR Placebo	24 weeks	Histology: N Imaging: ✓ (FibroScan) Biomarker: ✓ (AST, ALT, fasting insulin)	Mild fungal genitourinary infections, increased truncal fat mass
		Phase 2 NCT02686476 (E-LIFT) [60]	Adult NAFLD with T2DM 50	**10 mg/day** OR Placebo	20 weeks	Histology: N Imaging: ✓ (MRI-PDFF) Biomarker: ✓ (ALT)	
**Semaglutide**	GLP-1 analog	Phase 2 NCT02970942 [61]	Adult NASH	**0.4 mg/day** OR 0.2 mg/day OR 0.1 mg/day OR Placebo	72 weeks	Histology: ✓ Imaging N Biomarker: X	Mild gastrointestinal disorders
		Phase 3 NCT04822181 (ESSENCE)	Adult NASH with F2–F3 1200	Per week (dosage unknown) OR Placebo	72, 240 weeks	Pending (May 2028)	
**Liraglutide**	GLP-1 analog	Phase 2 NCT01237119 (LEAN) [62]	Adult biopsy-proven NASH	**1.8 mg/day** OR Placebo	48 weeks	Histology: ✓ Imaging: N Biomarker: ✓ (GGT)	Well-tolerated
**Metformin**	AMPK inhibitor	Phase 2 [63]	Adult biopsy-proven NAFLD without diabetes 55	**2 g/day** OR 800 IU/day Vitamin E OR Placebo with diet	12 months	Histology: ✓ Imaging: N Biomarker: ✓ (ALT, AST, HOMA-IR)	Well-tolerated
		Phase 2 [64]	Adult biopsy-proven NAFLD 48	**5 g/day** OR Placebo	6 months	Histology: X Imaging: N Biomarker: ✓ (glucose, HbA1c, cholesterol, LDL-C, body weight)	
		Phase 2 [65]	Adult biopsy-proven NAFLD without diabetes 19	**1 g/day** OR Placebo with weight loss program	12 months	Histology: X Imaging: N Biomarker: X	

Legend: dosage. **Bold** = Drug(s) evaluated in results. Results: ✓ = Significant compared to placebo/non-bolded drugs; X = Not significant compared to placebo/non-bolded drugs; N = No results available.

**Table 6:** Clinical trials on lipid-lowering drugs evaluated.

Lipid-Lowering Drugs							
Drug	Mechanism of Action	Trial (Type and Identifier)	Patient (Type and Number)	Dosage	Duration	Results (Histology, Imaging, Biomarker) ✓ X	Side Effects
**Ezetimibe**	NPC1L1 inhibitor	Phase 2 (UMIN000005250) [103]	Adult biopsy-proven NASH 32	**10 mg/day** OR Placebo	6 months	Histology: ✓ Imaging: N Biomarker: X	Worsened insulin resistance with increased risk of T2DM
**Statins**	HMG-CoA inhibitor	Phase 2 [106]	Adult biopsy-proven NASH 16	**Unspecified dosage** OR Placebo	12 months	Histology: X Imaging: N Biomarker: X	Well tolerated
**Aramchol**	SCD-1 inhibitor	Phase 2b (NCT02279524) [107]	Adult biopsy-proven NASH with prediabetes or T2DM 247	**600 mg/day** OR **400 mg/day** OR Placebo	52 weeks	Histology: X Imaging: ✓ Biomarker: ✓ (HbA1c)	Well tolerated
		Phase 3 NCT04104321 (ARMOUR) [108]	Adult NASH with F1-F3 150	300 mg/day OR Placebo	24, 48, 72, 96, 120 weeks	Pending (June 2027)	
**Oltipraz**	LXRα inhibitor	Phase 2 (NCT01373554) [23].	Adult US-proven NAFLD Asian 68	**120 mg/day** OR 60 mg/day OR Placebo	24 weeks	Histology: N Imaging: ✓ Biomarker: ✓ (BMI, HDL-C)	Well tolerated

Legend: dosage. **Bold** = Drug(s) evaluated in results. Results: ✓ = Significant compared to placebo/nonbolded drugs; X = Not significant compared to placebo/nonbolded drugs; N = No results available.

**Table 7:** Clinical trials on bile pathway drugs evaluated.

Bile Pathway Drugs							
Drug	Mechanism of Action	Trial (Type and Identifier)	Patient (Type and Number)	Dosage	Duration	Results (Histology, Imaging, Biomarker) ✓ X	Side Effects
**Obeticholic acid**	FXR agonist	Phase 3 NCT02548351 (REGENERATE) [122]	Adult non-cirrhotic biopsy-proven NASH, with F2–F3 or F1 with at least 1 comorbidity 2480 (931 F2–F3 patients in interim analysis)	**25 mg/day** OR 10 mg/day OR Placebo	18 months (interim analysis), 7 years (end of study)	Histology: ✓ (based on interim analysis, NASH resolution outcome not met yet) Imaging: N Biomarker: X	Significant pruritus (lead to rejection of accelerated approval pathway)
		Phase 2 (FLINT) [119]	Adult non-cirrhotic biopsy-proven NAFLD 283	**25 mg/day** OR Placebo	18 months	Histology: ✓ Imaging: N Biomarker: X	
		Phase 3 NCT03439254 [55]	Adult NASH 919	10 to 25 mg/day OR 10 mg/day OR Placebo	18 months	Pending	
**Cilofexor**	Selective non-bile acid intestinal FXR agonist	Phase 2b NCT03449446 (ALTAS) [123]	Adult biopsy-proven NASH, F3-F4 392	**30 mg/day** OR 18 mg/day selonsertib OR 20 mg/day firsocostat OR 30 mg/day with 18 mg/day selonsertib OR 30 mg/day with 20 mg/day firsocostat OR Placebo	48 weeks	Histology: X Imaging: X (measured by FibroScan) Biomarker: X (ALT, AST, bilirubin, cytokeratin-18, insulin) (All results were significant for cilofexor-firsocostat)	Cilofexor has the highest percentage of adverse events
		Phase 2 (NCT02854605) [62]	Adult non-cirrhotic biopsy-proven NASH 140	**100 mg/day** OR **30 mg/day** OR Placebo	24 weeks	Histology: ✓ Imaging: ✓ (via MRI-PDFF) Biomarker: ✓ (GGT, bile acids, C4)	Pruritus (especially in 100 mg/day group)
**EDP-305**	FXR agonist	Phase 2 [124]	Adult fibrotic NASH 134	**2.5 mg/day** OR 1.5 mg/day OR Placebo	12 weeks	Histology: N Imaging: ✓ (via MRI-PDFF) Biomarker: ✓ (ALT)	Pruritus

Legend: dosage. **Bold** = Drug(s) evaluated in results. Results. ✓ = Significant compared to placebo/non-bolded drugs; X = Not significant compared to placebo/non-bolded drugs; N = No results available.

**Table 9:** Clinical trials on other drugs evaluated.

Other Drugs							
Drug	Mechanism of Action	Trial (Type and Identifier)	Patient (Type and Number)	Dosage	Duration	Results (Histology, Imaging, Biomarker) ✓ X	Side Effects
**Resemtirom**	THR-β agonist	Phase 2 NCT02912260 [146]	Adult biopsy-proven non-cirrhotic NASH 348	**80 mg/day** OR Placebo	36 weeks	Histology: ✓ Imaging: ✓ (via MRI-PDFF) Biomarker: ✓ (ALT, AST, GGT, ELF, CK-18, adiponectin, LDL, triglycerides)	Well tolerated
		Phase 3 NCT03900429 (MAESTRO-NASH) [147]	Adult biopsy-proven NASH with F2–3 2000	100 mg/day OR 60 mg/day Or Placebo	52 weeks	Pending	
**NGM282**	FGF19 analog	Phase 2 NCT02443116 [148]	Adult biopsy-proven NASH	6 mg/day OR 3 mg/day OR Placebo		Histology: ✓ Imaging: ✓ (via MRI-PDFF) Biomarker: ✓ (ALT, AST, triglycerides, pro-C3, ELF)	Well tolerated
		Open label [149]	Adult biopsy-proven NASH	**3 mg/day** OR **1 mg/day** OR Placebo	12 weeks	Histology: ✓ Imaging: X Biomarker: X	
**Pegbelfermin**	FGF21 analog	Phase 2a NCT02413372 [150]	Adult biopsy-confirmed NASH 75	**20 mg/week** OR 10 mg/week OR Placebo	16 weeks	Histology: N Imaging: ✓ (via MRI-PDFF) Biomarker: X	Well tolerated
		Phase 2 (FALCON) [151]				Pending	
		Phase 2 (FALCON 2) [151]				Pending	
**Vitamin E**	Antioxidant	Phase 3 Random double blind NCT00063622 (PIVENS) [44]	Adult NASH 247	**800 mg/day** OR 30 mg/day pioglitazone OR Placebo	24 months	Histology: ✓ Imaging: N Biomarker: ✓ (ALT, AST)	Well tolerated
		Phase 2 [152]	Adult NASH 102	**800 mg/day** OR 500 mg/day UDCA OR 800 mg/day PTX	3 months	Histology: N Imaging: N Biomarker: ✓ (IL6, CCL2/MCP-1 AST, ALT)	
		Phase 2 [153]	Adult NASH 69	**800 mg/day with 1200 mg/day PTX** OR 800 mg/day	12 months	Histology: ✓ Imaging: X (via FibroScan) Biomarker: X	

Legend: dosage. **Bold** = Drug(s) evaluated in results. Results. ✓ = Significant compared to placebo/non-bolded drugs; X = Not significant compared to placebo/non-bolded drugs; N = No results available.
